# Advanced Dental Composite Technology via Bisilanized Dual‐Action Nanofillers for Biofilm Control

**DOI:** 10.1002/advs.75146

**Published:** 2026-04-07

**Authors:** Chenmin Yao, Line Etiennot, Naiera Zayed, Shengjie Liang, Mehraveh Saghi, Jelle Verdonck, Lingyue Liu, Fei Zhang, Wim Teughels, Cui Huang, Kirsten Van Landuyt, Bart Van Meerbeek

**Affiliations:** ^1^ KU Leuven, Department of Oral Health Sciences BIOMAT & UZ Leuven, Dentistry Leuven Belgium; ^2^ State Key Laboratory of Oral & Maxillofacial Reconstruction and Regeneration Key Laboratory of Oral Biomedicine Ministry of Education Hubei Key Laboratory of Stomatology School & Hospital of Stomatology Wuhan University Wuhan China; ^3^ KU Leuven, Department of Oral Health Sciences Periodontology & Oral Microbiology Leuven Belgium; ^4^ KU Leuven, Department of Public Health and Primary Care Environment and Health Leuven Belgium; ^5^ KU Leuven, Department of Chemical Engineering Soft Matter, Rheology and Technology (SMaRT) Leuven Belgium; ^6^ KU Leuven, Department of Materials Engineering Surface and Interface Engineered Materials (SIEM) Leuven Belgium

**Keywords:** bisilanization, dental caries, multimodal combined strategy, oral biofilm, rat tooth‐restoration model

## Abstract

Achieving durable tooth restorations in the complex oral environment demands resin‐based composites (RBCs) with long‐lasting antibiofilm properties, superior mechanical performance, and biosafety. This study introduces a duomodal strategy to create functional nanofillers for advanced RBC formulations. Mesoporous silica nanoparticles are loaded with the antibacterial agent cetylpyridinium chloride (CPC) and bisilanized using the antibacterial dimethyloctadecyl[3‐(trimethoxysilyl)propyl]ammonium chloride (DTSACl) along with 3‐(trimethoxysilyl)propyl methacrylate. The resulting bisilanized nanofillers, S_CM, are incorporated into RBCs at various weight percentages (0–20 wt%). The 20 wt% S_CM‐RBC formulation showcases exceptional antibiofilm capabilities against 14 oral species, including cariogenic, periopathogenic, and commensal bacteria, leveraging a dual antibacterial mechanism: CPC release and anchored DTSACl action. Additionally, S_CM nanofillers can disrupt bacterial fatty acid metabolism and ATP/nucleotide metabolism. Enhanced mechanical performance is achieved through superior filler–matrix coupling enabled by bisilanization. The formulation demonstrates low water sorption (< 40 µg mm ^−^^3^) over 3 months and high flexural strength (> 80 MPa). Antibiofilm activity and biosafety are further confirmed in an in vivo rat tooth‐restoration model. This dual‐antibacterial, bisilanized RBC offers promising clinical opportunities to durably restore teeth by effectively controlling biofilm, preventing caries recurrence, and reducing the risk of periodontal infections. The technology holds great potential for advancing restorative dentistry and promoting oral health.

## Graphical abstract

A multimodal research strategy has led to the development of an innovative resin‐based composite (RBC) with dual antibacterial action. The S_CM‐RBC formula showed strong antibiofilm activity, excellent mechanical strength, and biosafety. It effectively controlled oral bacteria in prevention of caries recurrence, and maintained pulp health in a rat model, offering a durable solution for longer lasting and healthier dental restorations.

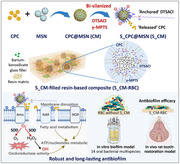

## 1 Introduction

Biofilms are a persistent challenge in both medicine and dentistry, playing a key role in chronic infections [1]. Defined as microbial communities embedded in extracellular polymeric substances [2], biofilms thrive on tooth surfaces, contributing to common oral diseases like dental caries and periodontal disease [3]. With the adult mouth spanning approximately 215 cm^2^, 20% of which are tooth surfaces [4], and harboring between 100 and 200 species of oral bacteria [5, 6], oral biofilms are readily formed on tooth surfaces. Alarmingly, oral microbes have also been detected in distant organ sites, such as the brain, lungs, heart, and placenta [7], highlighting the systemic implications of oral biofilm. Dental caries, the world's most prevalent disease, affects over two billion people globally [8]. While resin‐based composites (RBCs) have become the standard for restoring caries‐affected teeth due to their aesthetic appeal and ease of use [9], they remain vulnerable to secondary caries [10] and limited durability [11]. The persistent accumulation of biofilms on RBC surfaces poses significant challenges, accelerating restoration breakdown, promoting further tooth destruction toward ultimately premature tooth loss. This has driven an urgent demand for RBCs with enhanced therapeutic functionalities and improved stability. Developing advanced RBCs with long‐lasting antibiofilm properties and superior physicochemical performance is essential to extend restoration longevity and contribute to better oral and systemic health outcomes.

RBCs are primarily composed of inorganic fillers (60–85 wt%), resin monomers, and a photoinitiation system [12]. Recent advancements in RBCs focus on modifying fillers to enhance their therapeutic potential and mechanical performance. Porous fillers, in particular, have demonstrated the ability to improve filler–matrix interactions through micromechanical interlocking [13]. Among these, mesoporous silica nanoparticles (MSNs) have gained significant attention. MSNs consist of amorphous silica walls surrounding uniform mesopores, making them highly versatile for biomedical applications, particularly as drug‐delivery systems [14, 15]. As filler, silica‐based MSNs offer a refractive index compatible with resin matrices [16], which is crucial for maintaining superior optical and esthetical properties. MSNs are particularly promising for imparting antibiofilm/antimicrobial capabilities to RBCs due to their high specific surface area, ease of surface functionalization, and excellent biocompatibility [17, 18]. Encapsulation of antibacterial agents within MSNs enables a controlled and gradual antibacterial effect. As surface wear occurs, fresh MSN particles loaded with antibacterial agents are exposed, continuously releasing the agent and sustaining the antibacterial effect over time. However, ensuring effective antibacterial delivery while maintaining mechanical integrity and biosafety of RBCs remains a critical challenge. Addressing these factors is key to the successful development of advanced RBC technology.

Several antibacterial fillers, including silver, zinc oxide, and chlorhexidine, have been developed and play pivotal roles in combating microbial growth [12]. One major limitation of these releasing systems is the short‐lived burst release [19], which is followed by a significant decline in release rate, ultimately undermining their long‐term antibacterial effectiveness. Therefore, there remains a growing demand for long‐lasting and highly efficient antibacterial fillers. Cetylpyridinium chloride (CPC), a cationic quaternary ammonium salt (QAS), is a potent broad‐spectrum antibacterial agent with notable antiviral activity [20, 21]. QAS‐based materials have extensively been explored for their antimicrobial applications, primarily targeting the cell membrane as their site of action. MSNs provide a promising platform for delivering CPC, owing to their high surface area, which enables sufficient loading of antibacterial agents. By incorporating CPC, CPC‐loaded MSN (CPC@MSN, or CM filler) is anticipated to exhibit significant antibacterial efficacy. Furthermore, the silanol groups on the interior and exterior surfaces of MSNs offer excellent versatility for additional chemical modifications [22]. Combining CPC with additional antibacterial materials covalently bonded to these silanol groups can further enhance therapeutic efficacy. A quaternary ammonium silane dimethyloctadecyl[3‐(trimethoxysilyl)propyl]ammonium chloride (DTSACL), is particularly suitable for silanizing MSNs [23], as it introduces positively charged quaternary amine groups with complementary antibacterial properties. DTSACL can be combined with 3‐methacryloxypropyltrimethoxysilane (γ‐MPTS), a commonly used dental silane, to enable dual functionalization of MSNs. This dual silanization ensures robust mechanical properties while retaining a strong antibacterial effect. Upon light‐emitting diode irradiation, bisilanized CPC@MSN (S_CPC@MSN, or S_CM filler) copolymerizes with resin monomers through the vinyl groups provided by γ‐MPTS. This process enhances filler–matrix coupling via chemical binding, supplemented by micromechanical interlocking of resin within the MSN pores. However, encapsulated CPC may be constrained by the finite release of its active compound, limiting its effective duration, whereas the bactericidal efficacy of DTSACL contact‐killing surfaces can be reduced in a protein‐rich environment. Therefore, integrating encapsulated CPC with quaternary ammonium silane (DTSACL) could provide a synergistic approach to potentially enhance and prolong antibacterial performance.

**FIGURE 1:**
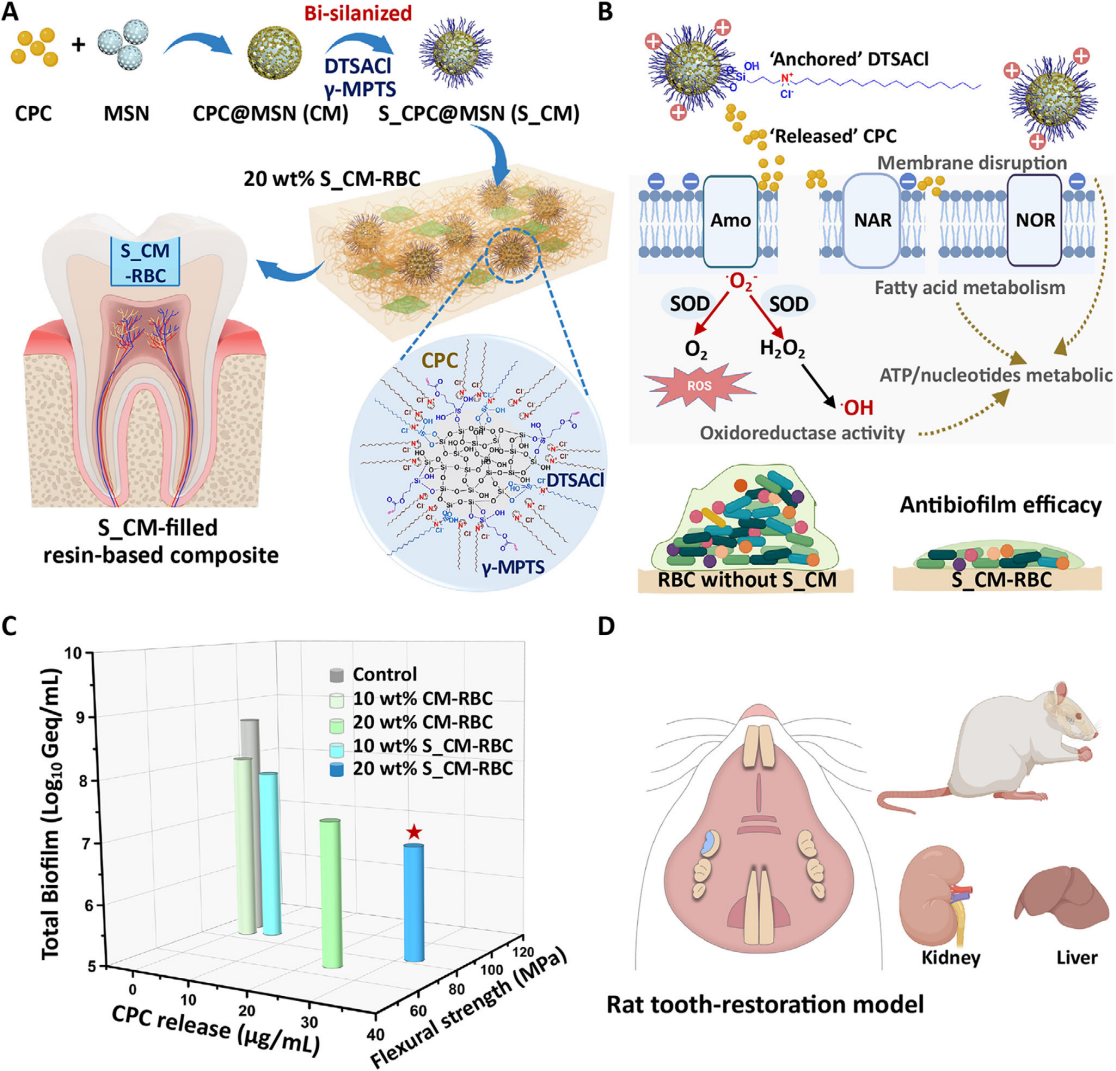
Design and fabrication of silanized CPC@MSN‐filled resin‐based composite (S_CM‐RBC). (A) Synthesis of S_CM‐RBC. (B) Proposed antibiofilm mechanism of S_CM‐RBC through generating reactive oxygen species (ROS), membrane disruption, affecting fatty acid metabolism, and ATP/nucleotides metabolism, synergistically produced by “released” CPC and “anchored” DTSACl (Amo: ammonium monooxygenase, NAR: nitrate reductase, NOR: nitrite oxidoreductase, SOD: superoxide dismutase). (C) Comparison of antibiofilm efficacy (with the total biofilm bacterial load data represented as log_10_ genome equivalents (Geq) per milliliter), CPC release, and flexural strength of the different RBCs investigated. The red star highlighted the 20 wt% S_CM‐RBC formulation, which exhibited effective antibiofilm activity while maintaining strong mechanical properties. Control: (S_)CM‐free control RBC. (D) Antibiofilm efficacy and biosafety of S_CM‐RBC validated in vivo using a rat tooth‐restoration model.

Building on these considerations, we developed experimental nanohybrid RBCs incorporating CM and bisilanized S_CM fillers, designated as CM‐RBC and S_CM‐RBC, respectively, to investigate their antibiofilm efficacy and physicochemical stability within the oral microenvironment (Figure 1A–C). The experimental RBCs were evaluated using an in vitro biofilm model comprising 14 prominent oral bacterial species, including two cariogenic species, four periopathogens, three anaerobic commensals, and five streptococcal commensals. The S_CM filler demonstrated effective bacterial attachment mediated by electrostatic interactions thanks to its positive charge. A synergistic antibacterial mechanism was observed, based on the release of CPC and the anchoring of DTSACl, which generated reactive oxygen species (ROS), disrupted the bacterial cell membranes, and affected fatty acid metabolism and ATP/nucleotide metabolism, ultimately inducing cell lysis. Notably, S_CM‐RBC containing 20 wt% S_CM filler (20 wt% S_CM‐RBC) exhibited effective antibiofilm activity in vitro while maintaining strong mechanical properties. Further validation in a rat tooth‐restoration model confirmed the robust and sustained antibiofilm effects of S_CM‐RBC in vivo, along with its outstanding biosafety profile (Figure 1D). These findings underscore the potential of S_CM‐RBC as a promising material for durable and therapeutic dental restorations.

## 2 Results and Discussion

### 2.1 Preparation and Characterization CM and S_CM

**FIGURE 2:**
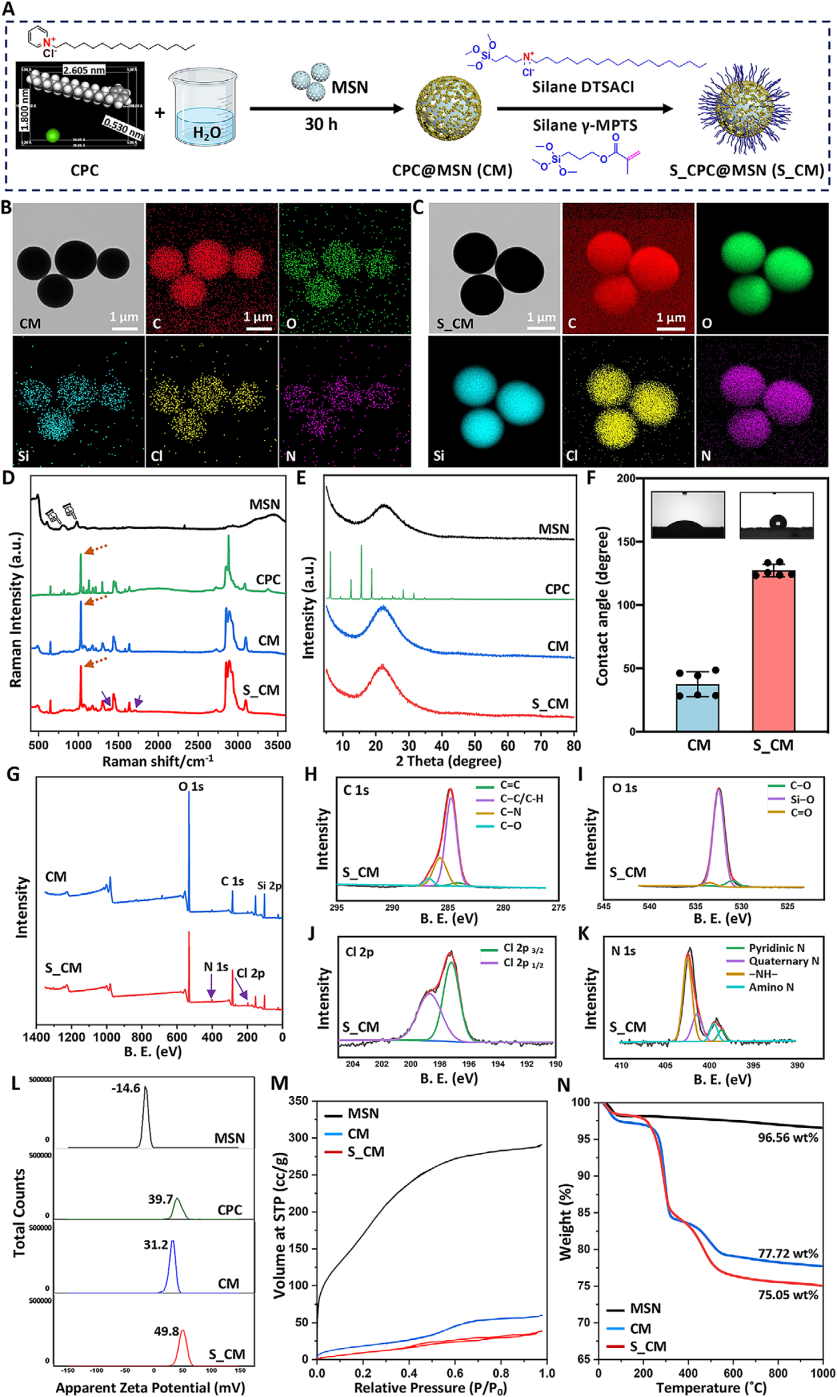
Structural and compositional characterization of CM and S_CM. (A) Schematic presenting CM and S_CM filler preparation. (B,C) HRTEM and EDS elemental distribution of CM and S_CM. (D) Raman spectra of MSN, CPC, CM, and S_CM. Characteristic peaks (black hand pointers) at 803.5 and 980 cm^−1^ within MSN, assigned to symmetric stretching vibrations of Si─O─Si bonds and Si─OH groups. The strong peak (orange dotted arrow) around 1030 cm^−1^ was attributed to the pyridine ring of CPC. After silanization, the peak (purple arrow) around 1420 cm^−1^ detected for S_CM should be assigned to the C═O and O─H groups of γ‐MPTS. Another C═O band (purple arrow) at 1715 cm^−1^ was detected for S_CM as well. (E) XRD patterns of MSN, CPC, CM, and S_CM. (F) Water contact angles of CM and S_CM. Data presented as mean with standard deviation (SD) (*n* = 6). (G) XPS spectra of CM and S_CM. (H–K) Deconvoluted C 1s, O 1s, Cl 2p, and N 1s peak of S_CM. (L) Zeta potential of MSN, CPC, CM, and S_CM. (M,N) Nitrogen adsorption/desorption isotherms and thermogravimetric analysis of MSN, CM, and S_CM.

Figure 2A illustrates the synthesis of CM and S_CM. The obtained CM was further functionalized with the potentially antibacterial silane DTSACl and conventional silane γ‐MPTS. High‐resolution transmission electron microscopy and energy‐dispersive X‐ray spectroscopy (HRTEM/EDS, Figure 2B,C) revealed the morphology of the CM and S_CM filler having a 750 to 1000 nm diameter. EDS mapping showed a uniform distribution of C, O, Si, Cl, and N within CM and S_CM. Cl and N were unique to CPC in CM, confirming that CPC was loaded into the MSN pores (Figure 2B). Micro‐Raman spectra revealed characteristic peaks at 803.5 and 980 cm^−1^ within MSN, respectively, assigned to symmetric stretching vibrations of Si─O─Si bonds and Si─OH groups (Figure 2D) [24]. The strong peak around 1030 cm^−1^ is attributed to the pyridine ring of CPC [25], and is still clearly detected for CM and S_CM. After silanization, the peak around 1420 cm^−1^ detected for S_CM should be assigned to the C═O and O─H groups of γ‐MPTS [26]. Another C═O band at 1715 cm^−1^ was detected for S_CM [27]. X‐ray powder diffraction (XRD) revealed a broad diffraction peak at 2*θ* = 23° [28], indicating the amorphous silica character of MSN and the developed CM and S_CM (Figure 2E). Furthermore, CM and S_CM presented water contact angles of, respectively, 37.5 ± 9.8° and 127.3 ± 5.0° (Figure 2F), demonstrating that silanization enhanced hydrophobicity.

X‐ray photoelectron spectroscopy (XPS) revealed C, O, Si, Cl, and N signals in wide‐scan XPS spectra of CM and S_CM (Figure 2G), confirming the abovementioned EDS data. The deconvoluted C 1s peaks appearing at 284.1, 284.7, 285.7, and 286.7 eV should be assigned to C═C, C−C and C─H, C─N, and C─O bonds of S_CM (Figure 2H), all bonds confirming MSN silanization [29, 30]. The O 1s peak was deconvoluted into a C─O peak at 531 eV, a Si─O peak at 532.5 eV, and a C═O peak at 533.4 eV (Figure 2I). The Cl 2p peak was fitted to the Cl 2p3/2 peak at 197.2 eV and the Cl 2p1/2 peak at 198.7 eV [31] (Figure 2J), indicative of Cl^−^ in S_CM. The N 1s signal demonstrated four N atom types for S_CM (Figure 2K): pyridinic N at 398.6 eV, quaternary N at 401.4 eV, ─NH─ at 402.5 eV, and amino N at 399.5 eV [32, 33], which were agreed with the pyridine ring of CPC and quaternary ammonium group of DTSACl. The average zeta potentials of MSN, CPC, CM, and S_CM were measured as −14.6, +39.7, +31.2, and +49.8 mV, respectively (Figure 2L), indicating a transition from negative to positive charges during the synthesis of S_CM. Initially, the MSN surface is characterized by negatively charged, hydrophilic silanol groups (Si─OH) [15], which facilitate electrostatic interactions with the cationic CPC molecules. Loading CPC into the MSN structure shifted the zeta potential from negative to positive, a change further intensified by dual silanization. This modification enhanced the formation of an electrostatic surface layer, promoting strong attraction to negatively charged bacterial cell membranes, thereby serving as an effective antibacterial mechanism [34]. N_2_‐adsorption/desorption confirmed the highly mesoporous structure of MSN, with the pore volume/size substantially decreasing upon CPC loading (CM) and silanization (S_CM) (Figure 2M). The specific surface area (*S*_BET_), pore volume, and pore size of MSN calculated by the Brunauer–Emmett–Teller/density functional theory (BET/DFT) method were 675.6 m^2^ g^−1^, 0.421 cc g^−1^, and 3.2 nm (Table S1). This initially high surface area of MSN implies a high potential for antibacterial drug loading. After loading CPC, the *S*_BET_ and pore volume of CM decreased to 72.0 m^2^ g^−1^ and 0.079 cc g^−1^, respectively. By silanization, the *S*_BET_ and pore volume of S_CM further decreased to 50.5 m^2^ g^−1^ and 0.026 cc g^−1^, respectively. Finally, the degree of CPC loading and silanization of S_CM were quantitatively analyzed using thermogravimetric analysis, as representatively shown in Figure 2N. The average amount of CPC uploaded within MSN was 18.2 wt%, and the average degree of CM silanization was 3.0 wt%.

### 2.2 Antibacterial Properties of CM, S_CM, and CM‐ and S_CM‐Filled Resin‐Based Composites (CM‐RBC and S_CM‐RBC)

**FIGURE 3:**
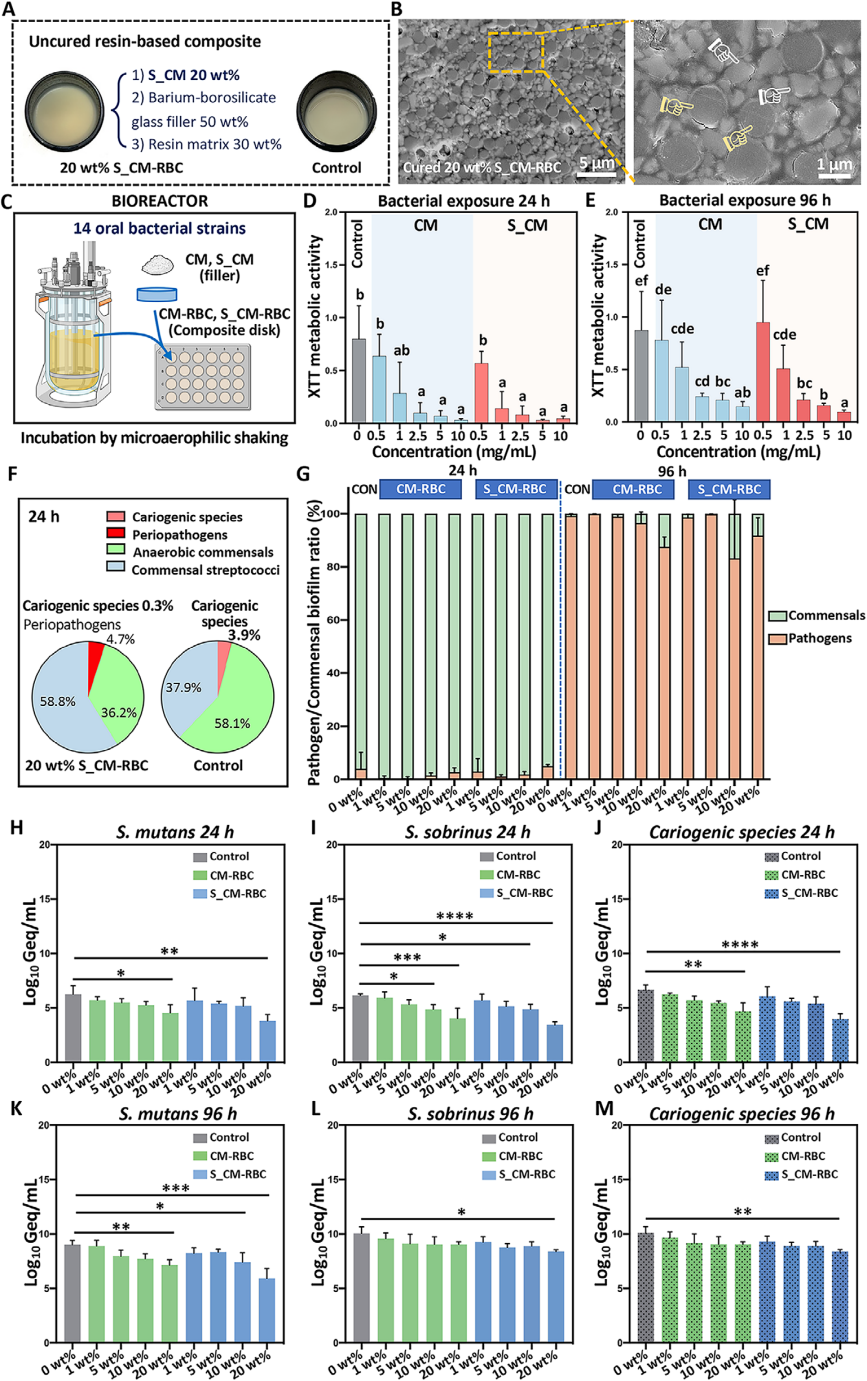
Antimicrobial efficacy of CM, S_CM, CM‐RBC, and S_CM‐RBC in vitro. (A) Preparation of experimental S_CM‐RBC. (B) SEM photomicrographs of cured 20 wt% S_CM‐RBC (white hand pointer: silanized barium‐borosilicate glass filler; yellow hand pointer: S_CM filler). (C) Preparation of 14 oral bacterial strains using the bioreactor. (D,E) 24 and 96 h XTT assay of CM and S_CM, with the data expressed as mean with SD and indicated by the error bars (same lower letters represent absence of statistical difference: *p* > 0.05, *n* = 3). Statistical analysis was performed using Welch's ANOVA followed by Games‐Howell multiple comparisons. (F) Proportional composition of the 24 h biofilm of 20 wt% S_CM‐RBC and (S_)CM‐free control RBC. (G) Distribution percentages of pathogens and commensals in biofilms. (H–J) 24 h antimicrobial efficacy of CM‐RBC and S_CM‐RBC on *S*. *mutans*, *S*. *sobrinus*, and cariogenic bacteria, as compared to (S_)CM‐free control RBC. (K–M) 96 h antimicrobial efficacy on *S*. *mutans*, *S*. *sobrinus*, and cariogenic bacteria. One‐way ANOVA with Dunnett's multiple comparisons test: *p < 0.05, **p < 0.01, ***p < 0.001, and ****p < 0.0001 (*n* = 3).

To prepare the experimental RBCs, CM or S_CM fillers at 1, 5, 10, and 20 wt% were individually mixed with conventional 0.7 µm silanized barium‐borosilicate glass filler and resin matrix (Table S2 and Figure S1). As illustrated in Figure 3A, the 20 wt% S_CM‐RBC contained 20 wt% antibacterial S_CM filler, 50 wt% barium‐borosilicate glass filler, and 30 wt% resin matrix, whereas the control RBC was composed solely of 70 wt% barium‐borosilicate glass filler and 30 wt% resin matrix. After curing, CPC embedded within the S_CM filler may be incorporated into the polymer network through hydrophobic interactions [20]. Scanning electron microscopy (SEM) images revealed a homogeneous dispersion of the spherical S_CM fillers and irregularly shaped barium‐borosilicate glass fillers throughout the experimental RBCs (Figure 3B).

To better mimic the natural oral microbiome compared to single‐specie models, an in vitro biofilm model comprising 14 representative oral bacterial species was applied, including cariogenic species, periopathogens, anaerobic commensals, and streptococcal commensals (Figure 3C; Table S3) [35, 36]. Among these, the gram‐positive *S*. *mutans* and *S*. *sobrinus* exhibit cariogenic potential, while the gram‐negative *A*. *actinomycetemcomitans*, *F*. *nucleatum*, *P*. *gingivalis*, and *P*. *intermedia* are recognized as periopathogens [6, 37, 38, 39]. Certain commensal bacteria, including streptococci and *Actinomyces* species, act as primary colonizers and play a critical role in adhesion and biofilm initiation [38, 40]. Sugars are a major factor in promoting cariogenic biofilms by disrupting the balance between commensals and opportunistic pathogens [4]. Notably, *S*. *mutans* converts sucrose into extracellular insoluble glucans, enhancing bacterial adhesion and forming the extracellular polymeric substance core [4]. To better simulate a cariogenic biofilm environment, 5% sucrose was added to the modified BHI medium (BHI‐2). The inhibitory effects of CM and S_CM fillers on bacterial growth were evaluated using an XTT assay. After 24 h exposure, bacteria cultured in the presence of 2.5 mg mL^−1^ CM exhibited significantly reduced metabolic activity compared to control in BHI‐2 with 5% sucrose (*p* < 0.05; Figure 3D). Even at a lower concentration of 1 mg mL^−^, S_CM achieved similar reductions in metabolic activity, attributed to the synergistic antibacterial effects of CPC and DTSACL silane. After 96 h biofilm formation, both CM and S_CM demonstrated concentration‐dependent reductions in metabolic activity (*p* < 0.05; Figure 3E). Notably, the lowest metabolic activity was observed with 10 mg mL^−1^ S_CM, underscoring its superior antibacterial efficacy.

The antimicrobial efficacy of CM‐RBC and S_CM‐RBC on oral biofilms was next evaluated using viability quantitative PCR (v‐qPCR) assays. First, the biofilm ecology and relative abundance of the 14 species within the biofilm ecosystem, were calculated to illustrate the community composition. The 24 h biofilms formed on all RBCs contained over 90% commensal species (anaerobic commensals and commensal streptococci) (Figure 3F,G; Figure S2), with the anaerobic commensal *Veillonella parvula* dominating the biofilms formed on the 20 wt% CM‐RBC, 20 wt% S_CM‐RBC and (S_)CM‐free control RBC (Figure S3). However, the proportion of commensals in the 96 h biofilms significantly declined to 3.5%, 8.2%, and 0.8%, respectively, for the 20 wt% CM‐RBC, 20 wt% S_CM‐RBC and control RBC (Figure S2). By 96 h, the cariogenic species *S*. *mutans* and *S*. *sobrinus* became dominant in the control biofilm, while the streptococcal commensals, primarily *S. sanguinis*, were still identified in the 10 and 20 wt% CM‐RBC, and 10 and 20 wt% S_CM‐RBC biofilms (Figure S3). These results demonstrated that the composites with higher concentrations of CM‐RBC and S_CM‐RBC prevented the comprehensive invasion of cariogenic species.

Looking at the absolute number of the cariogenic species in the biofilms, there was a significant reduction of *S*. *mutans* by 1.7 log_10_ genome equivalents per milliliter (Geq mL^−1^) with 20 wt% CM‐RBC in the 24 h biofilm, compared to the control (Figure 3H). A greater reduction of 2.5 log_10_ Geq mL^−1^ was observed with 20 wt% S_CM‐RBC. Similarly, for *S. sobrinus*, both 10 and 20 wt% CM‐RBC and S_CM‐RBC achieved significant reductions (Figure 3I). When the two cariogenic species were analyzed together (Figure 3J), reductions of 2.0 log_10_ Geq mL^−1^ and 2.7 log_10_ Geq mL^−1^ were observed for 20 wt% CM‐RBC and 20 wt% S_CM‐RBC, respectively, indicating the superior antibacterial efficacy of S_CM‐RBC. Both *S*. *mutans* and *S*. *sobrinus* are gram‐positive bacteria with teichoic acid layers in their cell walls [34]. These structural components likely interact with the positively charged S_CM, contributing to the high antibiofilm activity observed with 20 wt% S_CM‐RBC.

After 96 h incubation, only 20 wt% S_CM‐RBC demonstrated sustained performance against *S*. *mutans*, *S*. *sobrinus*, and their combined biofilm (Figure 3K–M). The enhanced long‐term antibiofilm efficacy of S_CM‐RBC compared to CM‐RBC can likely be attributed to the dual silanization of S_CM, which incorporates the antibacterial silane DTSACl. Following silanization, S_CM indeed exhibited a higher zeta potential than CM (Figure 2L), enabling it to neutralize the bacterial anion groups better and reduce bacterial adhesion on the composite surface. Both CPC and the silane DTSACl belong to the QAS family, with long alkyl chains (C16 in CPC and C18 in DTSACl). CPC loaded within MSN‐filler pores along with DTSACl silane chemically anchored to MSN‐filler surfaces in S_CM must synergistically have contributed to the long‐lasting antibiofilm activity. The long alkyl chains penetrate bacterial cell membranes, disrupting essential enzymes located on the inner cytoplasmic surface of the membrane [41, 42]. Key enzymes involved include ammonium monooxygenase (Amo), nitrite oxidoreductase (NOR), and nitrate reductase (NAR), which play roles in nitrification and denitrification processes [41, 43, 44].

**FIGURE 4:**
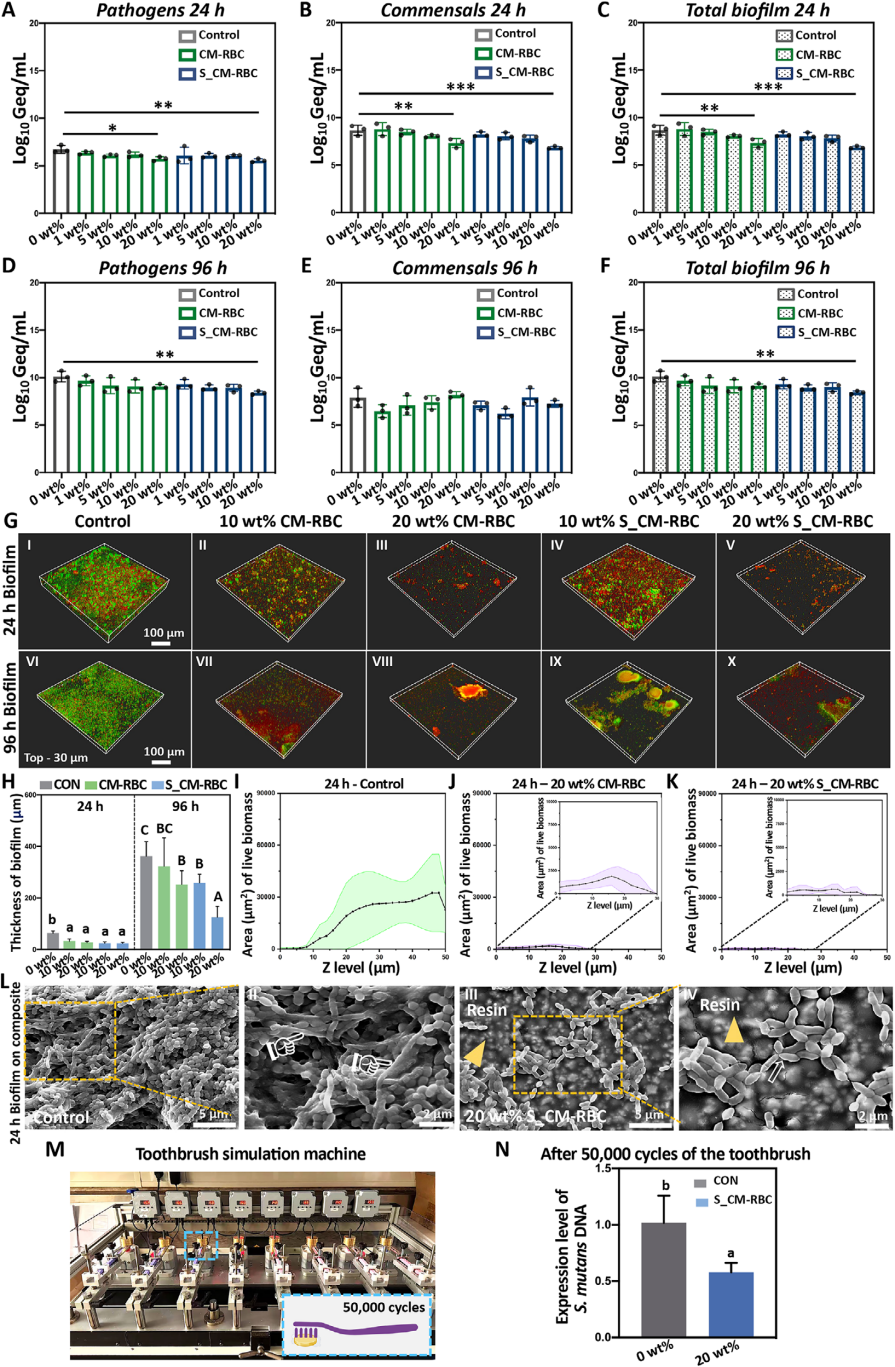
20 wt% S_CM‐RBC inhibits biofilm formation in vitro. (A–C) 24 h antimicrobial efficacy of CM‐RBC and S_CM‐RBC on pathogens, commensals, and total biofilm, as compared to the (S_)CM‐free control RBC. Data presented as mean ± SD. One‐way ANOVA with Dunnett's multiple comparisons test: *p < 0.05, **p < 0.01, and ***p < 0.001 (*n* = 3). (D–F) 96 h antimicrobial efficacy. (G) Representative CLSM images of live/dead bacterial staining of 14 oral bacterial strains on composite disks without (control) and with 10 and 20 wt% CM or S_CM fillers. After 24 h incubation, the full‐thickness biofilm is shown in (I)–(V)_._ After 96 h, the biofilm thickness increased, with solely the 30 µm biofilm top shown in (VI)–(X). (H) Biofilm thickness on CM‐RBC and S_CM‐RBC at 24 and 96 h, as compared to the (S_)CM‐free control RBC. One‐way ANOVA with Tukey's test. The same lower or upper letters represent absence of statistical difference: *p* > 0.05. (I–K) 24 h biofilm live biomass for the (S_)CM‐free control RBC, as compared to 20 wt% CM‐RBC and S_CM‐RBC. (L) SEM images of 24 h bacteria adhesion and biofilm deposition on composite disks for the (S_)CM‐free control RBC (I, II), as compared to 20 wt% S_CM‐RBC (III, IV). White hand pointer: rod‐shaped bacteria (likely pathobionts); white open arrow: cocci‐shaped bacteria (cariogenic pathogens or streptococci commensals); yellow triangle: resin‐based composite disk. The tapered end morphology of bacterial cells (IV) suggests membrane disruption and loss of cellular integrity. (M) Toothbrushing simulation machine. (N) Antibacterial assessment by qPCR, exhibiting expression levels of *S*. *mutans* DNA on resin‐based composite disks after 50,000 brushing cycles. Statistical analysis was performed using an unpaired two‐tailed Student's *t‐*test. Different lowercase letters indicate statistical difference (*p* < 0.05, *n* = 3). CON: S_CM‐free RBC.

Upon 24 h incubation, the bacterial populations of pathogens (including two cariogenic species and four periopathogens), commensals (including three anaerobic commensals and five streptococcal commensals), and the total biofilm were significantly reduced when exposed to 20 wt% CM‐RBC (*p* < 0.01) and 20 wt% S_CM‐RBC (*p* < 0.001) (Figure 4A–C). However, after 96 h incubation, only 20 wt% S_CM‐RBC demonstrated significant inhibition of pathogen growth and reduction in total biofilm compared to the control (*p* < 0.01, Figure 4D–F). This suggests that 20 wt% S_CM‐RBC possesses a more robust and durable antibacterial capability than 20 wt% CM‐RBC. To further assess the distribution of viable and nonviable bacteria, a live/dead bacterial fluorescence test was performed. For the 24 h biofilm of the control RBC, which lacked CM or S_CM fillers, pseudo‐3D confocal laser scanning microscopy (CLSM) revealed an abundance of green, viable bacteria growing on the composite surface (Figure 4G_I_: control). In contrast, 20 wt% CM‐RBC (Figure 4G_III_) and 20 wt% S_CM‐RBC (Figure 4G_V_) visibly inhibited bacterial growth. After 96 h incubation, the biofilm thickness increased, with the 30 µm biofilm top shown in the CLSM images of Figure 4G_VI–X_. For 20 wt% CM‐RBC (Figure 4G_VIII_) and 10 wt% S_CM‐RBC (Figure 4G_IX_), microcolonies with mushroom‐like structures were observed, indicating the formation of bacterial communities [45]. Compared to the control RBC, the biofilm densities of both 20 wt% CM‐RBC and 20 wt% S_CM‐RBC were notably reduced. Regardless of the incubation time (24 or 96 h), the biofilm thicknesses of 20 wt% CM‐RBC and 20 wt% S_CM‐RBC were significantly thinner than those of the control (Figure 4H), demonstrating superior resistance to biofilm formation. Furthermore, 20 wt% S_CM‐RBC exhibited a significantly 20‐fold lower live biomass than the control (Figure 4I–K). For the 24 h biofilm, SEM (Figure 4L; Figure S4) revealed for the control RBC a higher microbial load with rod‐shaped bacteria (likely pathobionts) and cocci‐shaped bacteria (cariogenic pathogens or streptococcal commensals). Conversely, 20 wt% S_CM‐RBC primarily displayed cocci‐shaped bacteria. SEM of the 96 h biofilm (Figure S5) confirmed thicker biofilms compared to the 24 h biofilms, consistent with the CLSM results (Figure 4H). Furthermore, toothbrushing is the most common oral hygiene approach and can be simulated in vitro to determine how dental materials perform in the long term [46]. The literature claims that 10,000 cycles are equivalent to 1 year of toothbrushing in the oral environment [47, 48]. After using the toothbrushing simulation machine for 50,000 brushing cycles (Figure 4M; Movies S1 and S2), 20 wt% S_CM‐RBC displayed reduced bacteria (Figure 4N), despite surface changes may result from pore clogging with toothpaste slurry. Although the toothbrushing simulation cannot fully replicate the real oral environment, it may provide relevant information and demonstrate the potential mid‐ to long‐term antibacterial performance of S_CM‐RBC. Overall, the microbial data consistently demonstrated the persistent inhibition of biofilm formation and accumulation by 20 wt% S_CM‐RBC.

### 2.3 Antibacterial Mechanism of Action

**FIGURE 5:**
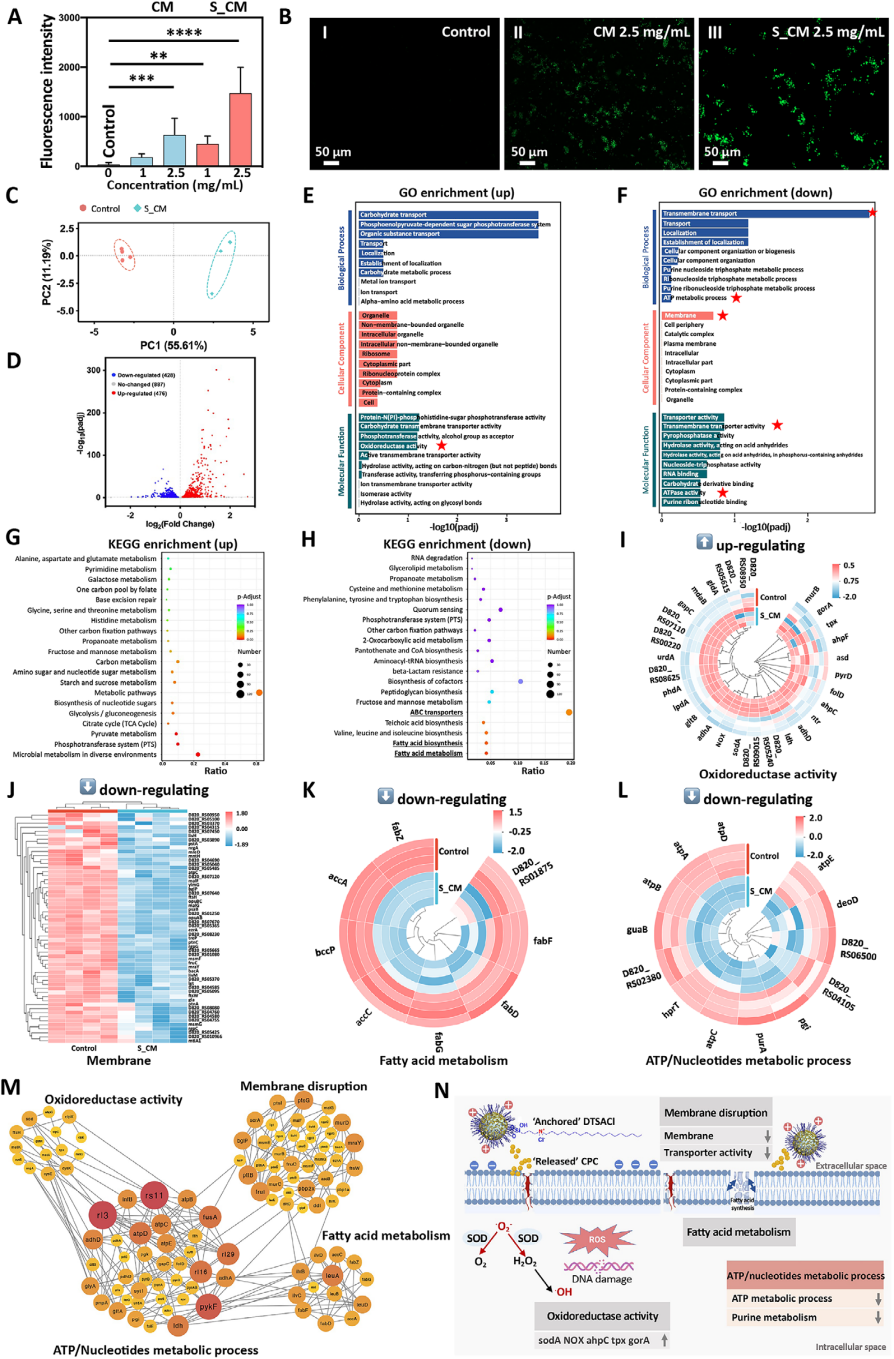
Intracellular ROS generation and RNA‐seq of *S*. *mutans* treated with S_CM. (A) Intracellular ROS expression of bacterial communities after CM and S_CM treatment based on green fluorescence intensity. Two biological replicates with five fields of view each were analyzed. One‐way ANOVA with Dunnett's multiple comparisons test: *p < 0.05, **p < 0.01, ***p < 0.001, and ****p < 0.0001. (B) The green fluorescence of fluorescent dichlorofluorescein (DCF) shows that 2.5 mg mL^−1^ S_CM treatment causes higher ROS production inside the bacteria. (C) Principal component analysis of *S*. *mutans* treated by control (PBS) and 2.5 mg mL^−1^ S_CM (*n* = 4). (D) Volcano map for the distribution of differentially expressed genes (DEGs). (E,F) GO enrichment analysis of upregulated and downregulated DEGs in the S_CM treated group compared to the control group. (G,H) KEGG enrichment analysis of upregulated and downregulated DEGs. (I–L) Heat map of the DEGs associated with oxidoreductase activity, membrane, fatty acid metabolism, and ATP/nucleotides metabolic process. (M) Protein–protein interaction network analysis of DEGs in the S_CM group. (N) Schematic mechanism of S_CM‐induced antibacterial performance.

The dichloro‐dihydro‐fluorescein diacetate (DCFH‐DA) probe was used to detect intracellular ROS generation. The intracellular ROS level is characterized by green fluorescence intensity of the stained bacterial communities. As shown in Figure 5A,B, the intracellular ROS level was substantially elevated in the S_CM group compared to the control, indicating a significant increase in S_CM. Furthermore, the intracellular ROS level of bacteria cultured on S_CM‐RBC and CM‐RBC is significantly higher than that of bacteria cultured on pure RBC disks, regardless of whether it is after 24 or 96 h (Figure S6). As reported, penetration of the hydrophobic alkyl chains induces excessive oxidative stress within bacterial cells [49]. Previous research has shown that QAS compounds impair the functions of superoxide dismutase (SOD) and catalase (CAT), two critical enzymes in the cellular defense against oxidative stress [41]. SOD converts superoxide radicals (O^2−^) into hydrogen peroxide (H_2_O_2_) or oxygen (O_2_) [50], while CAT breaks down H_2_O_2_ into water (H_2_O) and O_2_ [51]. When CPC and DTSACl inhibit SOD and CAT activity, ROS, including H_2_O_2_, O^2−^, and ·OH accumulate, leading to oxidative damage and bacterial cell death.

Furthermore, to comprehensively elucidate the antibacterial mechanism of S_CM on the biofilm‐associated transcriptional regulation, we conducted RNA transcriptomic analysis (RNA‐seq). Principal component analysis revealed alterations in *S*. *mutans* after S_CM treatment (Figure 5C). Compared with the control, the S_CM‐treated group exhibited 904 differentially expressed genes (DEGs), with 476 genes being upregulated and 428 genes being downregulated (Figure 5D). Gene ontology (GO) enrichment analysis indicated upregulation of oxidoreductase activity, organic substance transport, and phosphoenolpyruvate‐dependent sugar phosphotransferase system (Figure 5E; Figure S7), while transmembrane transport, ATP metabolic process, membrane, transmembrane transporter activity, and ATPase activity were downregulated (Figure 5F; Figure S7). The suppression of the ATP metabolic process and ATPase activity suggests impaired ATP synthesis, whereas downregulation of membrane and transmembrane transporter activity indicated disruption of bacterial membrane integrity. Meanwhile, Kyoto Encyclopedia of Genes and Genomes (KEGG) enrichment analysis (Figure 5G,H) showed that the DEGs were mainly involved in metabolic reprogramming, with downregulation of genes related to ATP‐binding ABC transporters, fatty acid biosynthesis, and fatty acid metabolism (Figure 5H). Key DEGs linked to oxidoreductase activity (*sodA*, *adhA*, *adhD*, *ahpC*, *tpx*, and *gorA*) [52, 53], membrane (*msmEFG*) [54], fatty acid metabolism (*fabZ*, *fabD*, *fabG*, and *accA*) [55], and ATP/nucleotides metabolic process (*atpA‐E*) [56] are shown in Figure 5I–L. We further investigated the association of DEGS in the above four pathways. Protein–protein interaction (PPI) network analysis (Figure 5M) demonstrated that S_CM treatment disrupted bacterial energy metabolism and macromolecule biosynthetic processes. Collectively, these findings (Figure 5N) suggest that S_CM exerts its antibacterial activity by enhancing oxidoreductase activity, compromising membrane integrity, and perturbing energy and metabolic homeostasis in bacteria.

### 2.4 Physicomechanical Properties of CM‐RBC and S_CM‐RBC

**FIGURE 6:**
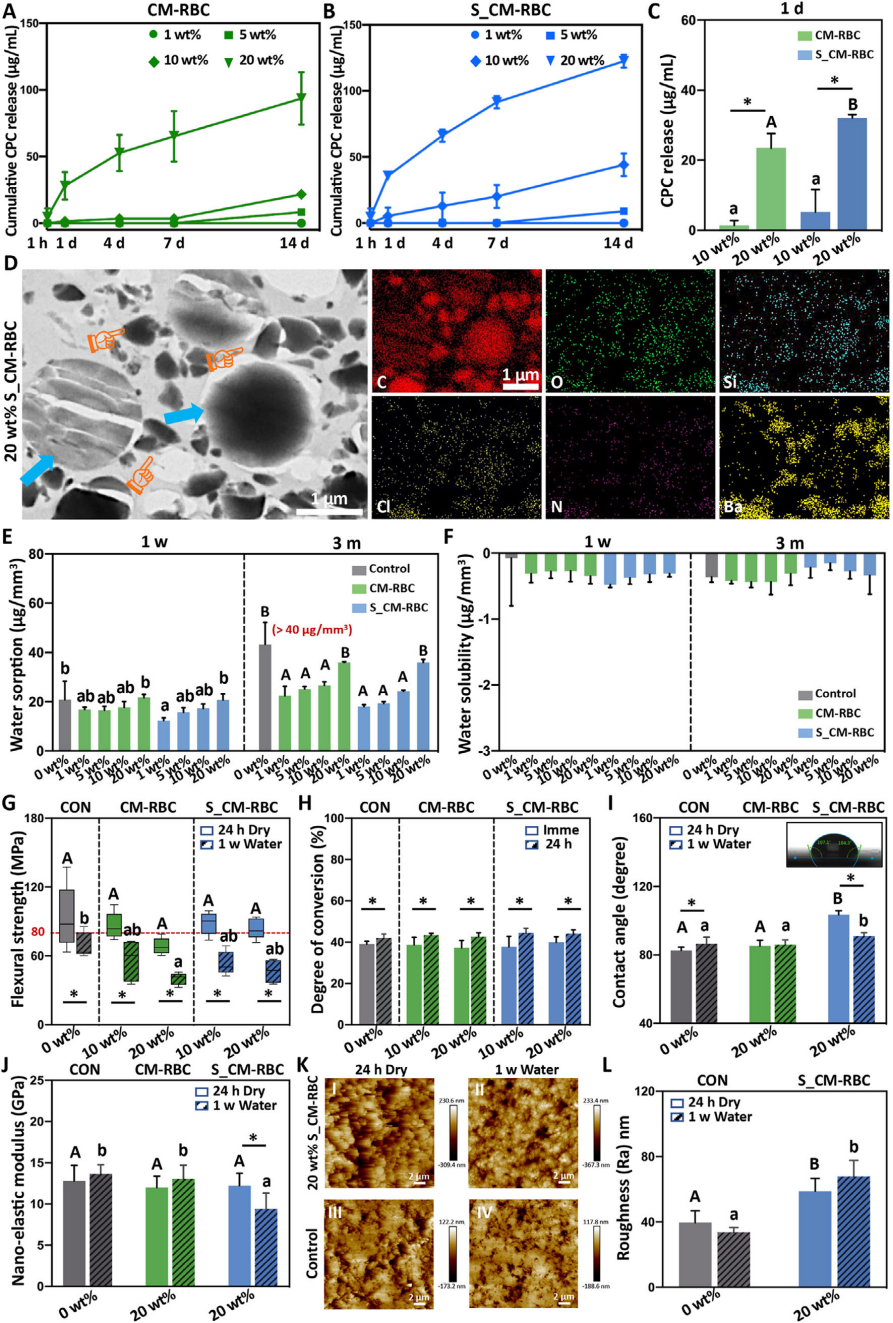
Physicomechanical properties of CM‐RBC and S_CM‐RBC formulations. (A,B) Cumulative CPC release from CM‐RBC and S_CM‐RBC formulations throughout a 14‐day period (*n* = 5). (C) CPC release from 10 and 20 wt% CM‐RBC and S_CM‐RBC, as measured after 1 day (same lower or upper letters indicate absence of statistical difference (*p* > 0.05); bars connected by a horizontal line with an asterisk indication are significantly different in pairwise comparisons (*p* < 0.05). (D) HRTEM/EDS of 20 wt% S_CM‐RBC (orange hand pointers: barium‐borosilicate glass filler; blue arrows: S_CM filler). (E) Water sorption after 1‐week and 3‐month water storage (*n* = 3). (F) Water solubility (*n* = 3). (G) Flexural strength. For the box‐and‐whisker plots, the boxes represent the first and third quartiles, and the whiskers denote the minimum and maximum (*n* = 5 per storage time). (H) Degree of conversion at two‐time intervals: immediate and 24 h post‐irradiation (*n* = 5). (I,J) Water contact angles (*n* = 6) and nanoelastic modulus (*n* = 3) of 20 wt% CM‐RBC and S_CM‐RBC, as compared to that of the (S_)CM‐free control RBC after 24 h dry storage and 1‐week water storage. The inset image in (I) was obtained from a 24‐h 20 wt% S_CM‐RBC sample. (K,L) Representative AFM topography photomicrographs and surface roughness (Ra, *n* = 3) of 20 wt% S_CM‐RBC and the (S_)CM‐free control RBC. Data presented as mean ± SD. Two‐way ANOVA (C,G–J,L) and one‐way ANOVA (E,F) were used for statistical analysis.

Combining ultra‐high‐performance liquid chromatography with mass spectrometry (UHPLC‐MS/MS) provides significant advantages in terms of sensitivity, selectivity, and high throughput for compound analysis [57]. Therefore, UHPLC‐MS/MS was used to detect CPC release from CM‐RBC and S_CM‐RBC throughout a 14‐day period. UHPLC‐MS/MS revealed a concentration‐dependent release from the experimental composites (Figure 6A,B). The highest amount of CPC was released from 20 wt% S_CM‐RBC. On day 1, the CPC‐release concentrations for 20 wt% CM‐RBC and S_CM‐RBC were 23.5 and 32.1 µg mL^−1^, respectively. 20 wt% S_CM‐RBC released significantly more CPC than 20 wt% CM‐RBC (*p* < 0.05, Figure 6C; Figure S8), indicating that CM‐filler silanization had a positive effect on CPC release. Previous studies reported that the charge expulsion between positively charged drugs and the particle surface enables the drugs to move more efficiently out of the pores [58]. As shown in Figure 2L, the zeta potential of S_CM (+49.8 mV) was higher than that of CM (+31.2 mV). Therefore, an increased charge expulsion promotes S_CM to release CPC more efficiently.

To further demonstrate the filler composition of S_CM‐RBC, its ultrastructure and elemental composition were characterized using HRTEM/EDS. HRTEM confirmed the homogeneous distribution of spherical S_CM and irregular barium‐borosilicate glass fillers within the experimental RBCs (Figure 6D). EDS mapping revealed that C, O, Si, and Ba were the main chemical elements. No significant difference in 1 week water sorption was measured between the CM‐ and S_CM‐filled RBCs as compared to the (S_)CM‐free control RBC (Figure 6E). The exception was 1 wt% S_CM‐RBC, exhibiting the significantly lowest water sorption. After 3 months of aging, water sorption of all CM‐RBC and S_CM‐RBC formulations remained below 40 µg mm^−3^, meeting the ISO 4049:2000 standard for water sorption by polymerized dental materials [59]. Although no significant difference in water sorption was recorded between 20 wt% CM‐RBC, 20 wt% S_CM‐RBC and the (S_)CM‐free control RBC, water sorption of the latter control reached 43.3 µg mm^−3^, hereby exceeding the abovementioned ISO standard. Adding CM or S_CM increased water sorption in a dose‐depending manner, with significantly most water sorption recorded for the 20 wt% (S_)CM‐filled RBCs. This suggests that the porous MSN filler may have acted as a pathway, facilitating water infiltration into the experimental (S_)CM‐filled RBCs. Notably, while 20 wt% S_CM‐RBC and CM‐RBC exhibited similar levels of water sorption, 20 wt% S_CM‐RBC released significantly more CPC than 20 wt% CM‐RBC (Figure 6A,B). This difference can be attributed to two factors. First, γ‐MPTS serves as an organic–inorganic bridging molecule that is covalently immobilized on the MSN surface and copolymerized with the polymer resin matrix. The cross‐linking effect of γ‐MPTS with the composite resin matrix creates structural barriers, reducing water penetration. Second, S_CM is inherently more hydrophobic, resulting in lower water sorption compared to CM. Additionally, the water solubility of all the experimental and control RBC revealed marginally negative values that remained below the ISO‐standard value of 7.5 µg mm^−3^ (Figure 6F).

RBCs are exposed to multiple forces in the complex oral environment, because of which their failure resistance should be evaluated. Regarding four‐point bending flexural strength [60], no significant differences were observed among the experimental and control RBC formulations after 24 h dry storage (Figure 6G). Their initial flexural strength was higher than 80 MPa, except for 20 wt% CM‐RBC (68.5 ± 7.1 MPa). The ISO‐4049 standard for polymer‐based restorations involving occlusal surfaces and upon intraorally curing specifies 80 MPa as the minimum initial flexural‐strength limit, as based on a three‐point bending test [61]. Notably, four‐point bending tests generally yield lower flexural strength values because they test a larger effective‐stressed volume of the material, exposing it to a greater likelihood of encountering imperfections. For polymer materials, which often have heterogeneities or microstructural defects, this difference is particularly noticeable. Therefore, the 10 and 20 wt% S_CM‐RBC, exhibiting an initial flexural strength above 80 MPa, meet the ISO‐standard limit, even when applied in four‐point bending, and theoretically could resist early fracture in the oral cavity. One week of water storage significantly decreased the flexural strength of all experimental and control RBC formulations, which should be ascribed to plasticization due to water sorption. The “immediate” degree of (polymerization) conversion (DC) of the experimental and control RBC formulations ranged between 37.3% and 40.0% after 20 s irradiation. It significantly increased after 24 h post‐irradiation to reach 42.0%–44.4% (*p* < 0.05, Figure 5H). No statistical differences in DC were found among the experimental and control RBC formulations, regardless of the measurement moment. Hence, the addition of CM and S_CM in 10 and 20 wt% concentrations did not affect resin polymerization. The contact angle of RBCs was evaluated regarding its potential influence on plaque formation. The 20 wt% S_CM‐RBC presented significantly higher water contact angles than the 20 wt% CM‐RBC, under both 24 h dry storage and one week of water‐storage conditions (Figure 6I), indicating that the filler silanization also effectively enhanced surface hydrophobicity. This finding is consistent with the observation that S_CM possesses greater hydrophobicity than CM (Figure 2F). Additionally, the 20 wt% S_CM‐RBC demonstrated superior and more sustained antibacterial capability compared to the 20 wt% CM‐RBC, suggesting that the increased contact angle may contribute to the inhibition of bacterial adherence.

The nanoelastic modulus of the materials was measured using nanoindentation. No significant differences in elastic modulus were observed among the experimental and control RBC formulations, whether assessed after 24 h dry storage or one week of water storage (*p* > 0.05, Figure 6J), with the exception of the significantly reduced nanoelastic modulus recorded for 20 wt% S_CM‐RBC. As previously mentioned, CPC release from S_CM‐RBC was significantly greater than that from CM‐RBC. Although the interfacial coupling between silanized CM particles (S_CM) and the resin matrix enhances the load‐bearing capacity and mitigates hydrolytic degradation, the reduced nanoelastic modulus of 20 wt% S_CM‐RBC may be attributed to its higher CPC release and water sorption. This finding suggests that concentrations exceeding 20 wt% S_CM may not be optimal.

The surface topography of the mirror‐polished 20 wt% S_CM‐RBC was analyzed and compared to the S_CM‐free RBC control using atomic force microscopy (AFM). Height images revealed a grainier topography for 20 wt% S_CM‐RBC (Figure 6K). This observation was further validated by surface roughness (Ra) measurements, which showed significantly higher Ra values for 20 wt% S_CM‐RBC than the control, regardless of the measurement time point (Figure 6L). Surface roughness above 200 nm (Ra) has been associated with increased bacterial accumulation and composite discoloration [62]. However, the highest Ra value for 20 wt% S_CM‐RBC in this study was 67.9 ± 9.9 nm, well below the 200 nm threshold. Overall, no adverse findings were identified for the experimental CM‐RBC and S_CM‐RBC formulations that might compromise their clinical performance in the challenging oral environment.

### 2.5 In Vitro Biosafety of CM‐RBC and S_CM‐RBC and In Vivo Biosafety of CM and S_CM

**FIGURE 7:**
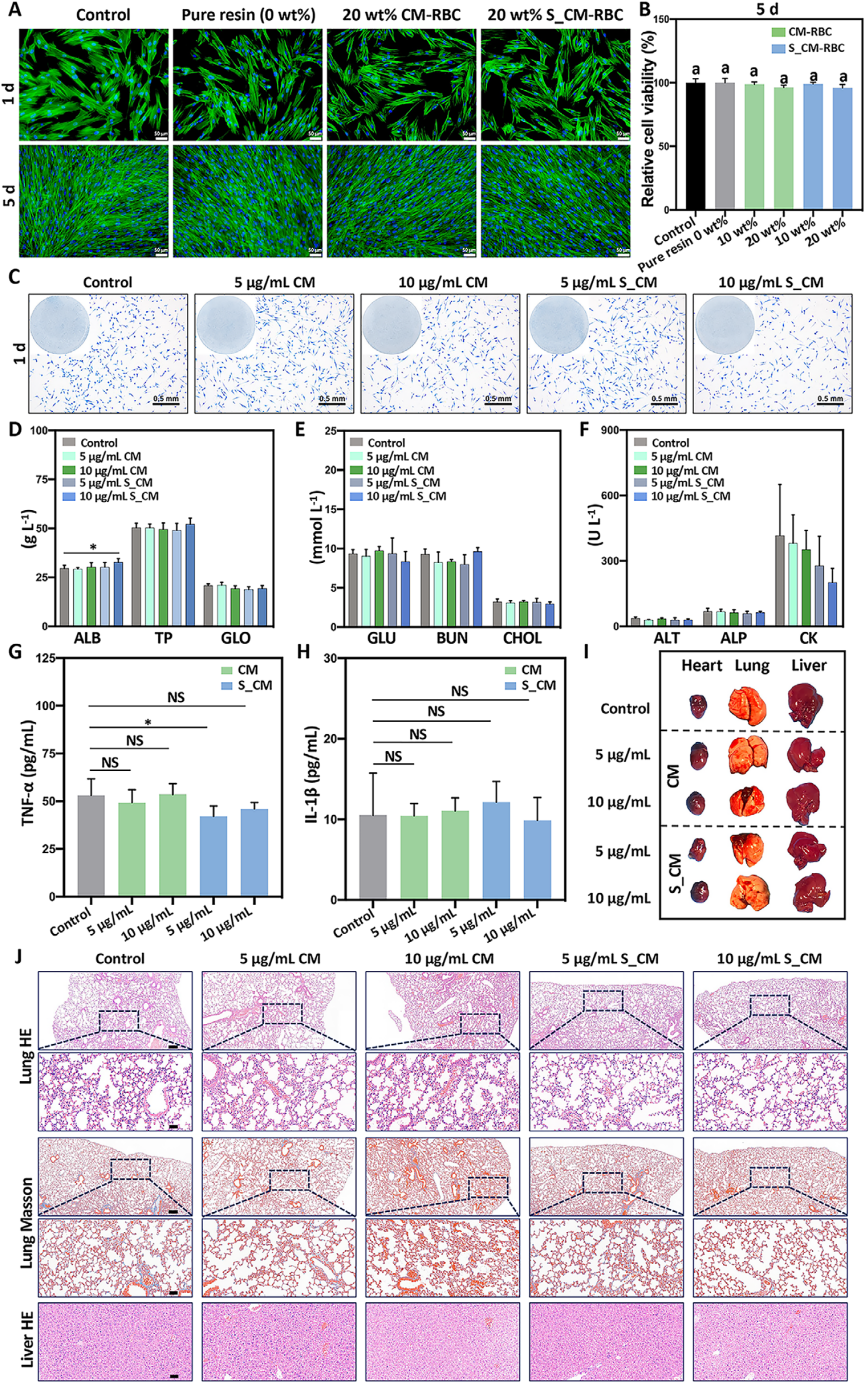
In vitro biosafety of CM‐RBC and S_CM‐RBC and in vivo biosafety of CM and S_CM. (A) Fluorescent labeling of the nucleus (blue) and actin (green) in hDPSCs at day 1 and day 5 after incubation with diluted RBC disk extraction [Blank control, pure resin 0 wt%: (S_)CM‐free RBC, 20 wt% CM‐RBC, and 20 wt% S_CM‐RBC]; scale bar: 50 µm. (B) Cell viability of hDPSCs cultured with RBC extraction for 5 days and analyzed by CCK‐8 assay. Data presented as mean ± SD. One‐way ANOVA with Tukey's test. The same lower letters represent absence of statistical difference: *p* > 0.05 (*n* = 3). (C) Coomassie blue staining of hDPSCs cultured with CM and S_CM at different concentrations (5 and 10 µg mL^−1^; blank control; scale bar: 0.5 mm). (D–F) Mice blood biochemistry examination at 2 weeks postinjection (*n* = 5). (G,H) Levels of proinflammatory cytokine TNF‐α and IL‐1β in sera from mice with CM and S_CM at different concentrations. One‐way ANOVA with Dunnett's multiple comparisons test. NS: no significance; *p < 0.05 (*n* = 5). (I) Representative photomicrographs of the heart, lung, and liver of mice. (J) Histological examination of lungs with HE and Masson's trichrome staining and liver with HE staining from mice with CM and S_CM (scale bars: 200 µm at low magnification and 50 µm at high magnification).

While MSN filler particles demonstrate low cytotoxicity in various in vitro cell‐line cultures [63], surface‐modified MSNs possess distinct surface chemistries that require careful biosafety evaluation [22]. CPC is known to exert toxic effects at high concentrations [64]. Ensuring the biocompatibility of CM‐RBC and S_CM‐RBC alongside effective antibacterial properties is critical for biomedical applications. Preserving tooth pulp vitality is a key step in managing deep caries [65]. The primary function of human dental pulp stem cells (hDPSCs) is dentin repair; these cells elicit a biological response when a tooth is restored with a restorative material. To evaluate the impact of CM‐RBC and S_CM‐RBC on hDPSCs attachment and proliferation, cytoskeleton staining was performed after 1 and 5 days of cell culture (Figure 7A; Figure S9). No significant differences in cell morphology or proliferation were observed between the experimental 20 wt% CM‐RBC, 20 wt% S_CM‐RBC, pure resin without (S_)CM filler, and the blank control. Additionally, a cytotoxicity assessment using a cell‐counting kit‐8 (CCK‐8) confirmed that 10 and 20 wt% CM‐RBC and S_CM‐RBC, did not adversely affect cell viability (Figure 7B; Figure S10).

The biosafety of CM and S_CM fillers was evaluated both in vitro using hDPSCs and in vivo through biological interaction studies. To assess their effects on hDPSCs, filamentous actins were stained with Coomassie blue. As shown in Figure 7C, well‐stretched filamentous actins were observed for both 10 µg mL^−1^ CM and S_CM, indicating that dual silanization did not adversely affect cell morphology or proliferation. Moreover, as previously reported, the toxicity of QAS on aquatic organisms decreases with longer alkyl chain lengths exceeding 14 carbon atoms [66]. With alkyl chain lengths of C16 for CPC and C18 for DTSACl, DTSACl is suggested to be less toxic than CPC. Additionally, the cytotoxicity of MSNs has been associated with their surface‐silanol groups, which can nonspecifically bind to cell‐membrane proteins, leading to cell lysis and necrosis [67]. However, the silanization process, in which DTSACl and γ‐MPTS covalently bond to MSN via silanol, reduces the density of surface‐silanol groups [68]. This creates a protective layer, minimizing interactions of silanols with cell‐membrane proteins. The in vivo biosafety of CM and S_CM fillers assessed in mice via a single intraperitoneal administration at concentrations of 5 and 10 µg mL^−1^ resulted in a 100% survival rate over two weeks. Potential cytotoxicity to normal tissues was further evaluated using serum biochemical parameters (Figure 7D–F). Among the parameters tested, only the albumin (ALB) level was significantly higher in the 10 µg mL^−1^ S_CM group compared to the control. No significant differences were observed for total proteins (TP), globulin (GLO), glucose (GLU), blood urea nitrogen (BUN, an indicator of kidney function), total cholesterol (CHOL), alanine aminotransferase (ALT, an indicator of liver function), alkaline phosphatase (ALP), or creatine kinase (CK). These findings demonstrate that CM and S_CM fillers did not cause significant damage to kidney or liver function or induce other systemic toxicities, supporting their biosafety for biomedical applications.

Both proinflammatory cytokines, tumor necrosis factor (TNF‐α) and interleukin‐1β (IL‐1β), are known to play a role in the progression of inflammatory processes [69]. In this study, the TNF‐α levels in the sera of mice treated with 5 µg mL^−1^ S_CM were significantly lower than those in the control group (α‐MEM with 10% fetal bovine serum (FBS) and 1% penicillin/streptomycin) (*p* < 0.05, Figure 7G). Furthermore, no significant differences in IL‐1β levels were observed among the CM, S_CM groups at various concentrations, and the control (Figure 7H). These results suggest that CM and S_CM fillers do not induce an acute inflammatory response. As shown in Figure 7I and Figure S11, histological examination of major organs, including the heart, lungs, liver, kidneys, and spleen, revealed no apparent signs of inflammation or tissue damage across all experimental and control groups. Additionally, histological analysis of the lungs and liver using hematoxylin and eosin (HE) and Masson's trichrome staining (Figure 7J; Figure S12) confirmed the absence of inflammatory cell infiltration or pathological alterations in mice treated with CM or S_CM, as compared to the control group. Together, these findings demonstrate that CM and S_CM fillers are biocompatible and do not elicit acute inflammatory responses, both in vitro and in vivo, hereby supporting their potential for safe use in biomedical applications.

### 2.6 In Vivo Antibacterial Performance and Biosafety of S_CM‐RBC in a Rat Tooth‐Restoration Model

**FIGURE 8:**
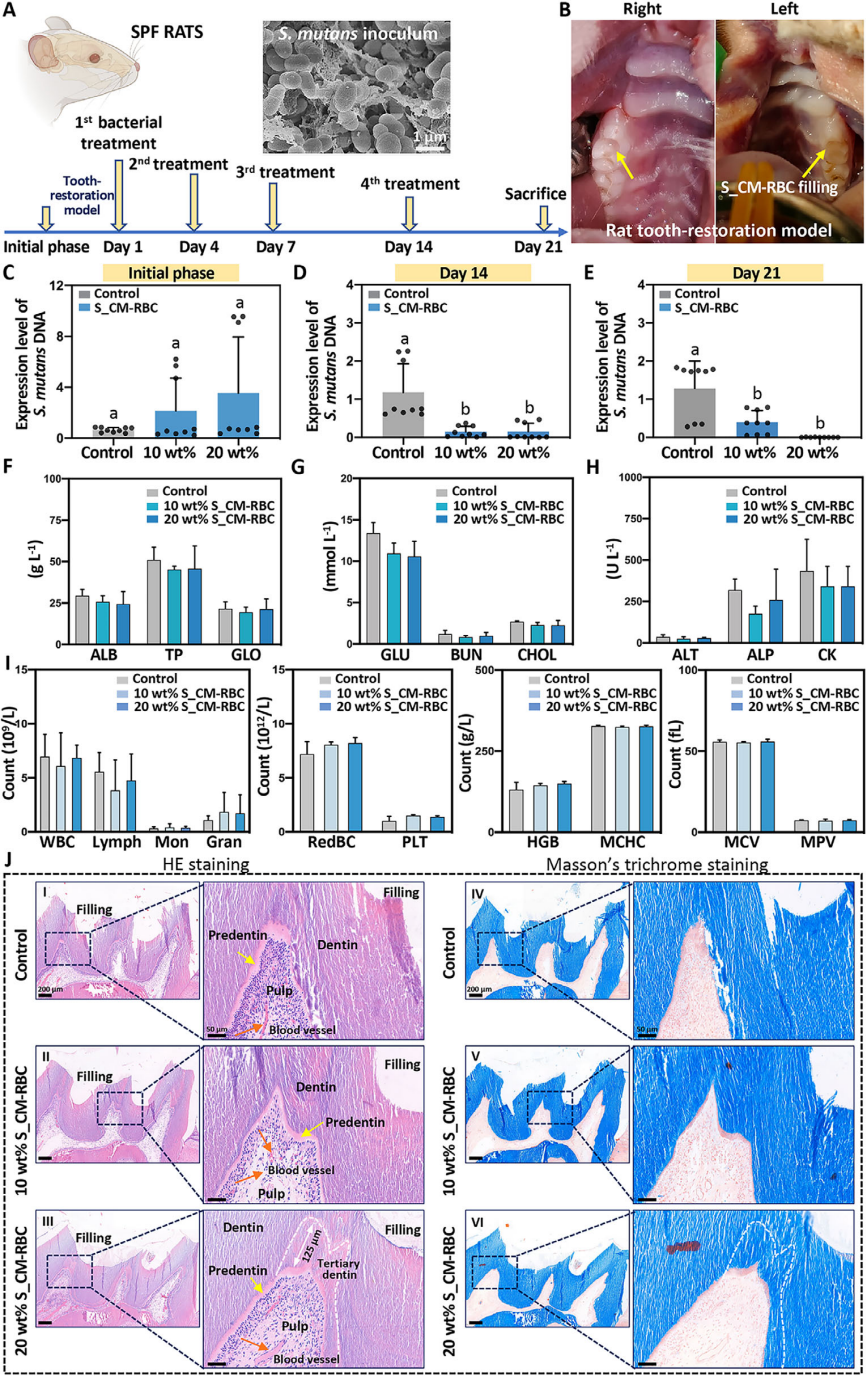
Antibacterial capability and biosafety of CM‐RBC and S_CM‐RBC in vivo. (A) Schematic illustration of the experimental design (SPF: specific‐pathogen‐free conditions). (B) Rat tooth‐restoration model. Occlusal cavities were prepared in the upper first molars and restored with experimental S_CM‐RBC. (C–E) Antibacterial assessment by qPCR, exhibiting expression levels of *S*. *mutans* DNA in rat teeth in the initial phase, and at day 14 and day 21. Same lower letters indicate the absence of statistical difference (*p* > 0.05). Control: (S_)CM‐free RBC. (F–H) Biochemistry examination of rat blood. (I) Routine rat‐blood examination. Data presented as mean ± SD (*n* = 3). Welch's ANOVA followed by Games‐Howell multiple comparisons (C–E) and one‐way ANOVA with Tukey's test (F–I) were used for statistical analysis. (J) Histological analyses of rat‐tooth cavities with HE and Masson's trichrome staining for the (S_)CM‐free control RBC, and the experimental 10 and 20 wt% S_CM‐RBC at day 21 (scale bars: 200 µm at low magnification and 50 µm at high magnification). Yellow arrows refer to the predentin structure. Orange arrows indicate blood vessels. A layer of tertiary dentin was found after the rat‐tooth cavity was filled with 20 wt% S_CM‐RBC.

Although many studies have shown significant reductions in biofilm formation or alterations in biofilm structures in the laboratory, few have been validated in vivo to evaluate their clinical significance [70]. Inspired by the superior in vitro antibacterial activity and biosafety of S_CM‐RBC, the antibacterial performance against *S*. *mutans* was studied in vivo. Sprague‐Dawley rats were used to establish a rat tooth‐restoration model (Figure 8A,B). Rat molar teeth can be considered as miniature human molars from a histological, biological, and physiological viewpoint. After initially prescreening the rats for *S*. *mutans*, tooth cavities with a 1 mm width and depth varying between 0.8 and 1 mm (verified using a periodontal probe) were prepared in the maxillary right and left first molars, and filled with the 10, 20 wt% S_CM‐RBC and the (S_)CM‐free control RBC formulations. Starting on day 1, the rats were orally infected with *S*. *mutans*, being the primary cariogenic oral microorganism and biofilm producer [37]. To prevent adverse effects of continuous anesthesia, the rats were inoculated with *S*. *mutans* suspensions on day 4, day 7, and day 14. As shown in Figure 8C, no significant differences in the expression levels of *S*. *mutans* DNA were initially observed among the rat teeth. On day 14 before the fourth infection and on day 21, viable bacteria were collected from the restorations in the maxillary first molars and analyzed using qPCR. The 10 and 20 wt% S_CM‐RBC restorations revealed significantly less *S*. *mutans* than the control restorations (*p* < 0.05, Figure 8D,E), confirming the efficient antibacterial capability of S_CM‐RBC in a rat tooth‐restoration model. To assess biocompatibility, also the blood biochemistry was examined. No significant differences were observed for the diverse parameters, including ALB, TP, GLO, GLU, BUN, CHOL, ALT, ALP, and CK, among the experimental and control RBC formulations (Figure 8F–H). This finding demonstrates that the application of 10 and 20 wt% S_CM‐RBC did not cause abnormalities in blood biochemistry and is safe for kidney/liver metabolism. Furthermore, routine blood examination (Figure 8I) showed no observable difference in the hematologic analytes, including the number/amount of white blood cells (WBC), lymphocytes (Lymph), monocytes (Mon), neutrophil granulocytes (Gran), red blood cells (RedBC), platelets (PLT), hemoglobin (HGB), mean corpuscular hemoglobin concentration (MCHC), mean corpuscular volume (MCV), and mean platelet volume (MPV). Notably, WBC count is a parameter that demonstrates whether a robust proinflammatory response is induced [71]. Additionally, PLT is essential for clotting and modulating immune responses against invading pathogens and particulate matter [72]. From the current results, WBC was in the normal range of 3.0–9.2 (10^9^ L^−1^), and PLT was in the normal range of 923–1580 (10^9^ L^−1^) [73]. This outcome confirmed the good biocompatibility of 10 and 20 wt% S_CM‐RBC when applied in vivo in blood.

The exceptional biocompatibility of S_CM‐RBC with the dentin–pulp complex is crucial for its potential biomedical application. To evaluate this, maxillae were collected after sacrificing the rats, upon which the maxillary first molars were histologically examined to assess the inflammatory conditions in pulp tissue. Representative histological photomicrographs of HE and Masson's trichrome‐stained sections of teeth following cavity restoration are presented in Figure 8J. In this study, deep cavities were prepared in the first molars, leaving a remaining dentin thickness of 100–200 µm. Teeth restored with 10 wt% S_CM‐RBC and control RBC exhibited normal pulp tissues and distinct predentin structures (Figure 8J–I,II), consistent with the healthy pulp conditions observed in the blank control (non‐restored second molar, Figure S13). Interestingly, 20 wt% S_CM‐RBC resulted in the production of a layer of tertiary dentin formed by odontoblasts and dental stem cells within the pulp tissue [74]. This tertiary dentin, deposited as new protective dentin, showed healing and regeneration capability (Figure 8J(III)). On day 21, a 125 µm thick tertiary dentin layer was observed, indicating moderate deposition of tertiary dentin (grade 2: 100–249 µm) [75]. This tertiary dentin barrier helps maintaining pulp vitality. Furthermore, Masson's trichrome staining confirmed the presence of reparative tertiary dentin in the 20 wt% S_CM‐RBC group (Figure 8J(VI)). These findings confirm that 20 wt% S_CM‐RBC restorations do not adversely affect the dentin–pulp complex in vivo. Additionally, HE images of gingival tissues (Figure S14) showed no visible histopathological inflammation or tissue damage, confirming that the experimental RBC treatment did not affect the adjacent soft tissues. Altogether, S_CM‐RBC offers a promising potential for use in biomedical applications. Nonetheless, additional preclinical assessments and further clinical trials are required to confirm biosafety. Meanwhile, SPF rats were used to enable controlled in vivo investigations. However, the model's limited microbial diversity and environmental complexity constrain direct extrapolation to human oral health. Thus, the current findings were interpreted as preliminary, requiring validation in more representative models before extrapolation to humans.

## 3 Conclusion

In summary, a multifunctional nanofiller S_CM was developed by integrating the antibacterial agent CPC and the QAS antibacterial silane DTSACl with the polymerizable silane monomer γ‐MPTS. The incorporation of S_CM into a resin matrix created the therapeutic composite S_CM‐RBC. A biofilm model of 14 critical oral bacterial species revealed that 20 wt% S_CM‐RBC delivered efficient antibiofilm activity. S_CM contributes to the disruption of bacterial cell membranes and affects fatty acid metabolism and ATP/nucleotide metabolism. The inclusion of γ‐MPTS played a key role in enhancing the interfacial coupling of the nanofiller with the polymer matrix, ensuring the stable mechanical properties of S_CM‐RBC. In vivo results further confirmed effective biofilm eradication and favorable preliminary biosafety. Overall, the dual‐antibacterial and bisilanized S_CM‐RBC represents a promising restorative material strategy for combating oral biofilms and extending the durability of dental restorations, making it a potential alternative for advanced therapeutic applications.

## 4 Experimental Section

### 4.1 Materials

MSNs (particle size: 750 nm) were obtained from Glantreo (Cork, Ireland). CPC, bisphenol A glycerolate dimethacrylate (BisGMA), triethylene glycol dimethacrylate (TEGDMA), camphorquinone (CQ), ethyl‐4‐dimethylaminobenzoate (EDMAB), cyclohexane, 2,3‐bis‐(2‐methoxy‐4‐nitro‐5‐sulfophenyl)‐2H‐tetrazolium‐5‐carboxanilide (XTT), and phosphate‐buffered saline (PBS), acetonitrile (ACN, LC‐MS grade), methanol (MeOH, LC‐MS grade), and formic acid (FA, LC‐MS grade) were purchased from Sigma Aldrich. γ‐MPTS was sourced from TCI EUROPE. DTSACl and *n*‐propylamine were supplied by Thermo Fisher Scientific. Barium‐borosilicate glass (0.7 µm, 1.5 wt% silanization) was acquired from Esschem Europe (Seaham, UK). A live/dead bacterial viability kit was purchased from Molecular Probes (Invitrogen, Eugene, OR, USA). LC‐MS grade water was purchased from Biosolve (Dieuze, France). Alpha‐MEM (α‐MEM) and penicillin/streptomycin were purchased from HyClone and Amresco LLC. Proinflammatory cytokine TNF‐α and IL‐1β assay kits were obtained from Elabscience Biotechnology Co., Ltd.

### 4.2 Preparation and Characterization of CM and S_CM

The MSNs were initially dried at 60°C overnight to eliminate moisture residues. The dried MSNs (10 mg mL^−1^) were subsequently dispersed in deionized water containing CPC (10 mg mL^−1^). The resulting mixture was subjected to sonication for 30 min, followed by vigorous stirring at 400 rpm for 28 h and shaking for an additional 2 h. The obtained CM was then centrifuged at 4350 rpm, washed with deionized water, and subsequently dried in an oven at 60°C. Silanization of the CM was performed by combining 5.0 g of CM, 0.1 g of *n*‐propylamine, and 0.5 g of silane (a 1:1 ratio of two types of silane: 0.25 g DTSACl and 0.25 g γ‐MPTS) in a 250 mL flask containing 100 mL of cyclohexane. The mixture was stirred continuously at room temperature for 30 min, then maintained at 65°C for 30 min. The mixture was subsequently subjected to moderate vacuum using a rotary evaporator at 50 ± 5°C until the solvent and volatile byproducts were effectively removed (approximately 40 min). The silane‐modified powder (S_CM) was finally obtained after drying overnight at 60°C.

The morphologies of CM and S_CM were observed using HRTEM (JEM‐1400 Flash, Jeol, Tokyo, Japan) at 120 kV. Elemental analysis of CM and S_CM was conducted via an EDS detector (Ultim Max, Oxford Instruments, Abingdon, UK). To confirm the incorporation of CPC and silane into MSN, micro‐Raman spectroscopy (Senterra, Bruker, Ettlingen, Germany) was performed using a near‐infrared laser with a wavelength of 532 nm and an output power of 20 mW. Pure CPC, MSN, CM, and S_CM were separately deposited on a transparent glass slide (with a powder weight of 5 mg). Spectral data were collected over a range of 400–3500 cm^−1^ with a resolution of 3–5 cm^−1^. The Raman measurements utilized a pinhole aperture of 50 µm, with an integration time of 20 s for the recorded spectra, which were averaged over three successive measurements. Five spectra were recorded per specimen to establish reproducibility. The XRD patterns of MSN, CPC, CM, and S_CM were characterized using Xpert Pro (Malvern Panalytical, The Netherlands) with Cu‐Kα radiation within a sweep range of 5° to 80°. The hydrophilic and hydrophobic properties of CM and S_CM were assessed through contact angle measurements, which were recorded via a Dataphysics OCA20 contact angle goniometer (DataPhysics Instruments GmbH, Filderstadt, Germany).

XPS analysis of the CM and S_CM was conducted via an ESCALAB250Xi spectrometer (Thermo Fisher Scientific, UK). The narrow‐scan XPS spectra of C 1s, O 1s, Cl 2p, and N 1s were deconvoluted with XPSpeak software. The zeta potential of MSN, CPC, CM, and S_CM was measured with a Malvern Zetasizer Nano ZSP at 25°C, and 12 zeta runs were conducted for each specimen (*n* = 3). To determine the surface area, pore size, and pore volume of MSN, CM, and S_CM, N_2_ adsorption–desorption isotherms were collected via a gas adsorption analyzer (Autosorb 1, Quantachrome, FL, USA) at 77 K. Prior to measurement, the specimens were degassed under vacuum at 40°C for 12 h. The silica surface area was calculated using the BET method, utilizing the adsorption data within a relative pressure range of 0.05–1. The pore‐size distribution was calculated from the desorption branch via the DFT method. To determine the CPC loading capacity, silanization, and thermal stability of the MSN, thermogravimetric analysis (SDT Q600, TA Instruments, New Castle, DE, USA) was carried out at a heating rate of 10°C/min, ranging from 25 to 1000°C under a nitrogen atmosphere.

### 4.3 Preparation of CM‐RBC and S_CM‐RBC

BisGMA and TEGDMA were mixed at a 1:1 ratio (14.85 wt%:14.85 wt%), followed by adding 0.06 wt% CQ and 0.24 wt% EDMAB. CM and S_CM were incorporated at 0, 1, 5, 10, and 20 wt% and blended with conventional 0.7 µm silanized barium‐borosilicate glass filler to achieve a total filler loading of 70 wt%. The component proportions within the experimental S_CM‐RBC formulations are detailed in Table S2. All the components were blended in a SpeedMixer (Hauschild SpeedMixer, Waterkamp, Germany). The resulting resin‐based composite paste was stored in a dark environment at room temperature for 4 h to allow adequate infiltration of the monomers into the MSN mesopores. Following curing with an LED light‐curing unit (SmartLite Pro, Dentsply Sirona, with an output of 1250 mW/cm^2^), morphological images of 20 wt% S_CM‐RBC were captured using SEM (FEI‐Nova Nanosem 450, FEI; Eindhoven, The Netherlands) with a 7.5 kV acceleration voltage, after a thin platinum/palladium (Pt/Pd) (Q150T S, Quorum tech, UK) coating was applied.

### 4.4 Bacterial Strains and Culture Conditions

Multiple species of oral communities were targeted, including 14 oral bacterial strains [35, 36], as outlined in Table S3. Bacteria were cultured on blood‐agar plates (Oxoid, Basingstoke, UK) supplemented with 5 µg mL^−1^ hemin, 1 µg mL^−1^ menadione, and 5% sterile horse blood. *Aggregatibacter actinomycetemcomitans* (*Aa*), *Fusobacterium nucleatum* (*Fn*), *Porphyromonas gingivalis* (*Pg*), *Prevotella intermedia (Pi*), *Actinomyces naeslundii* (*An*) and *Actinomyces viscosu (Av*) were incubated at 37°C under anaerobic conditions (10% CO_2_, 10% H_2_, 80% N_2_), whereas the remaining bacterial strains were incubated at 37°C in a 5% CO_2_ environment. Bacteria were collected from blood agar plates, transferred into 10 mL of brain heart infusion broth (BHI, Becton Dickinson and Company, Franklin Lakes, NJ, USA), and incubated overnight at 37°C under anaerobic conditions or a 5% CO_2_ environment.

### 4.5 Multispecies Communities and Biofilms

Multispecies bacterial communities were grown in a Biostat‐B Twin Bioreactor (Twin 1L, Sartorius Stedim Biotech, Goettingen, Germany) using modified BHI (BHI‐2) [35]. This modified BHI comprises BHI, porcine stomach type III mucin, yeast extract, l‐cysteine, sodium bicarbonate, and l‐glutamic acid.

### 4.6 Antibacterial Ability of CM and S_CM Assessed by XTT

A total volume of 300 µL of bacterial suspension (240 µL of BHI‐2 modified with 5% sucrose media + 30 µL of experimental material + 30 µL of bioreactor community) was inoculated into the wells of a 48‐well plate to facilitate the growth of a 14‐species biofilm. The final concentrations of CM and S_CM were adjusted to 0.5, 1, 2.5, 5, and 10 mg mL^−1^. Wells containing only the culture medium served as the control. The plates were incubated for either 24 or 96 h at 37°C under microaerophilic conditions (6% O_2_, 7% CO_2_, 7% H_2_, 80% N_2_) with shaking at 170 rpm. The culture medium was refreshed every 24 h to support bacterial growth. After incubation, the supernatant was discarded, and the wells were gently washed twice with PBS. The metabolic activity of CM and S_CM powders was evaluated via the XTT assay in conjunction with the cofactor phenazine methosulphate (PMS). An aliquot of 200 µL of the XTT‐PMS mixed solution (comprising 1 mg mL^−1^ XTT and 0.383 mg mL^−1^ PMS at a ratio of 50:1) was added to the wells. Following 3 h of incubation at 37°C [76], the plates were centrifuged at 8000 rpm for 3 min. Subsequently, 100 µL of the supernatant was added to a 96‐well microplate, and the absorbance was read at 450 nm (with a reference wavelength of 630 nm) via a Thermo Multiskan Ascent plate reader (ThermoFisher, Asse, Belgium). The experiment was performed in triplicate.

### 4.7 Biofilms on the Experimental CM‐RBC and S_CM‐RBC

Biofilms were cultivated on the surface of composite disks (diameter: 8 mm; thickness: 2 mm) placed in 24‐well plates. Composite disks (*n* = 3 per group) were prepared via custom‐made Teflon molds. The resin disk was light‐cured using the LED curing unit (SmartLite Pro) from the center for 30 s, followed by another 30 s of curing on the bottom after removal from the mold. Finally, the disks were light‐cured for 5 s on each of the four sides to ensure optimal curing, resulting in a total light‐curing time of 80 s. The surfaces not exposed to biofilms were marked. The specimens were cleaned with distilled water for 1 min and sterilized with UV light [77] for 15 min on each side. The bioreactor community was diluted at a ratio of 1:10 (v:v) in fresh BHI‐2 modified with 5% sucrose, after which 2 mL of this mixture was added to each well containing a resin‐composite disk. The plates were incubated for 24 or 96 h at 37°C under microaerophilic conditions with shaking at 170 rpm. The bacterial growth medium was renewed every 24 h without disturbing the established biofilm.

### 4.8 Biofilm Collection, DNA Extraction, and v‐qPCR Assay

The biofilms were detached from the resin‐composite disk using 0.05% trypsin‐EDTA (Life Technologies, Paisley, UK) for 45 min under anaerobic conditions at 37°C. The bacterial cells were collected by centrifugation (5 min at 6000 × *g*) and subsequently resuspended in 500 µL of PBS. A volume of 90 µL of the detached biofilms was exposed to propidium monoazide xx (PMAxx, Biotium, Hayward, CA, USA, 10 µL/well). The samples were kept in the dark for 10 min, after which PMAxx was photoactivated for 30 min using a Glo‐Plate blue (Biotium, Hayward, CA, USA). After photolysis, the samples were centrifuged for 10 min, and the supernatants were removed. According to the manufacturer's protocol, DNA from the pelleted cells was extracted with the QIAamp DNA Mini‐kit (Qiagen, Hilden, Germany). All viability quantitative PCR (v‐qPCR) assays were conducted on a CFX96 real‐time system (Bio‐Rad, Hercules, CA, USA). Specific details regarding the primer/probe and v‐qPCR components are listed in Tables S4 and S5.

### 4.9 CLSM of Live/Dead Bacterial Staining

After incubation, the biofilm‐coated specimen (at 24 and 96 h) was gently rinsed three times with PBS. All the specimens [(S_)CM‐free control RBC, 10 and 20 wt% CM‐RBC, and 10 and 20 wt% S_CM‐RBC] were subsequently placed into new 24‐well plates with the biofilm surface facing upward. The experimental resin‐composite disks were used for a live/dead bacterial staining test. Green and red fluorescence was obtained after staining live and dead bacteria with SYTO‐9 (*λ*ex = 488 nm) and propidium iodide (*λ*ex = 552 nm) for 15 min, and a live/dead bacterial viability kit was used. All specimens were observed using CLSM (Leica TCS SP8, Leica Microsystems GmbH, Wetzlar, Germany) with a resolution of 1024×1024 pixels (581×581 µm) and scans were conducted along the *Z*‐axis from the base of the biofilm (adjacent to the resin material). For the 24 h specimen, image stacks were acquired from four different locations (three triangle vertices that were equally distanced, close to the outer rim of the circle, and one at the center) with a 2 µm resolution in the *Z*‐axis. For the 96 h specimen, stacks were obtained from the same locations with a 10 µm *Z*‐axis resolution to accommodate the extreme thickness. In addition, a top 30 µm thick biofilm (positioned away from the resin material) was also recorded with a 2 µm resolution. The 3D build‐up overlay images were generated using Imaris software [78]. The biomass distribution of live bacteria in the 24 h biofilm was analyzed with Fiji ImageJ (National Institutes of Health, Bethesda, MD, USA). The CLSM tests were independently repeated twice [35].

### 4.10 Biofilm Ultrastructure by SEM

After incubation at 37°C for 24 or 96 h, adherent biofilm development on the RBC disks (*n* = 2 per group) was observed using SEM (FEI XL30‐FEG; FEI, Hillsboro, OR) with a 10 kV acceleration voltage. The disks with attached bacteria were rinsed twice with sterile PBS and fixed in 2.5% glutaraldehyde in 0.1 m sodium cacodylate buffer at pH 7.4 for 30 min. Following PBS washing to eliminate residual fixative, biofilms were dehydrated in ascending ethanol concentrations. After drying, disks were sputter‐coated with Pt/Pd 80/20 coating (Q150T S, Quorum tech, Laughton, UK).

### 4.11 Antibacterial Ability After Brushing Cycles

The experimental S_CM‐RBC and S_CM‐free control (CON) RBC (*n* = 3, diameter: 8 mm, height: 2 mm) were brushed for 50,000 cycles using a toothbrush simulation machine (SD Mechatronik, Feldkirchen‐Westerham, Germany), with a total brushing duration of 20 days. Each specimen was fixed on a sample holder that was kept in a plastic container filled with a slurry consisting of toothpaste (Colgate Total Active Prevention, Colgate‐Palmolive, Hamburg, Germany) and distilled water (using a paste‐to‐water ratio of 1:1.6) [79]. The toothpaste slurry was replaced daily. The toothbrushes (CS 3960 Soft, Curaprox, Swiss Premium Oral Care, Curaden, Kriens, Switzerland) followed a Z‐shaped path at a brushing speed of 25 mm/s, applying a 150‐g force in accordance with ISO 11609:2017 [80]. The brushed specimens were rinsed with water and dried before the antibacterial measurement. The 24 h biofilms were detached from the resin‐composite disk. DNA extraction was performed with a bacterial DNA kit according to the manufacturer's instructions. qPCR was performed with ChamQ Universal SYBR qPCR Master Mix (Nanjing Vazyme Biotech, Nanjing, China). Relative quantification and fold changes were evaluated by the 2^−ΔΔCt^ method. Universal primers specific for bacterial 16S rRNA genes (V1‐3 primers: 27F AGAGTTTGATCCTGGCTCAG and 534R ATTACCGCGGCTGCTGG) [81] and *S*. *mutans* primers (targeting the *gtfB* gene) were synthesized by Wuhan Tianyi Huiyuan Bioscience & Technology (Wuhan, China).

### 4.12 Determination of ROS Generation

Intracellular ROS production was evaluated using a ROS Assay Kit (Beyotime, China). Fourteen‐species bacterial communities (1 mL) were centrifuged and resuspended in 1 mL of PBS containing CM or S_CM (1 or 2.5 mg mL^−1^) for 20 min. The control was prepared by resuspending the bacteria in 1 mL of pure PBS. Samples were stained with 10 µm dichloro‐dihydro‐fluorescein diacetate (DCFH‐DA) at 37°C for 20 min in the dark to load the fluorescence probe [82]. Additionally, bacterial communities were cultured on S_CM‐RBC, CM‐RBC, and pure RBC surface. After 24 and 96 h, composite disks were rinsed twice with PBS, incubated with 600 µL PBS containing 10 µm DCFH‐DA for 20 min, and imaged at 20× magnification using a light microscope (Axio Imager M2, Carl Zeiss Microscopy, Jena, Germany). The images were processed with Fiji ImageJ (National Institutes of Health).

### 4.13 Transcriptomic Analysis (RNA‐seq)

*S*. *mutans ATCC 25175* (2 mL) was centrifuged and resuspended in 2 mL PBS containing S_CM (2.5 mg mL^−1^) and incubated for 6 h. Following treatment, bacteria were centrifuged and flash‐frozen in liquid nitrogen for RNA‐seq [83]. Total RNA was extracted, and high‐quality RNA was used for library construction. Sequencing was performed on an Illumina NovaSeq platform (paired‐end, 150 bp; NovaSeq‐PE150, Novogene Biotech Co., Ltd., China). Reads were quantified with FeatureCounts (v2.0.6), and differential expression analysis was conducted using DESeq2 (v1.42.0), based on Fragments Per Kilobase of transcript per Million mapped reads. GO and KEGG enrichment analyses were performed for bioinformatics interpretation [84].

### 4.14 CPC Elution From the RBCs by UHPLC‐MS/MS

The 1, 5, 10, and 20 wt% CM‐RBC and S_CM‐RBC disks (*n* = 5 per group) with a 5.5 mm diameter and 2 mm thickness were prepared via Teflon molds. The resin composite disks were light‐cured at the center from the top for 20 s. After removal from the mold, the bottom was cured for an additional 20 s. Finally, each disk was light‐cured for 5 s on all four sides to achieve optimal curing, resulting in a total light‐curing time of 60 s. After polymerization, the specimens were weighed and immersed (*n* = 5 per group) in glass vials containing 1 mL of LC‐MS grade water [85]. After immersion at 37°C, the corresponding solutions were collected at intervals of 1 h, 24 h, 4 days, 7 days, and 2 weeks. When the eluted solution was collected, refreshed water was added. The samples were stored at −20°C until analysis. All sample analyses were conducted via a Waters Micromass Quattro Premier mass spectrometer (Waters, Milford, MA, USA), equipped with electrospray ionization. Samples (10 µL) were introduced into a Waters Acquity UHPLC BEH C18 column (50 mm×2.1 mm, 1.7 µm). The mobile phase consisted of FA (0.1%) in H_2_O (eluent A) and FA (0.1%) in ACN (eluent B). Gradient elution was programmed as follows: 0 min, 5% B; 0.5 min, 5% B; 2 min, 50% B; 5 min, 100% B; 6 min, 5% B; 8 min, 5% B [86]. The mobile‐phase flow rate was 0.4 mL/min. During the analysis, the ion at *m*/*z* 304.3 [86] was identified as corresponding to the structure of CPC.

### 4.15 Ultrastructural HRTEM/EDS Characterization of S_CM‐RBC

Cured 20 wt% S_CM‐RBC was embedded in epoxy resin using a silicon mold. Upon storage for 18 h in a 60°C oven, TEM sections (100–120 nm) were cut using an ultramicrotome (Leica EM UC7, Vienna, Austria) and observed by HRTEM JEM‐1400 Flash (Jeol, Tokyo) at 120 KV. Element analysis of the S_CM‐RBC was performed with the EDS detector (Ultim Max).

### 4.16 Water Sorption and Water Solubility of RBC Formulations

Water sorption and solubility were measured by weighing RBC disks (1, 5, 10, and 20 wt% CM‐RBC and S_CM‐RBC, and control) after 7 days (*n* = 3) and 3 months (*n* = 3) of water immersion. Composite disks with a 15 mm diameter and 1 mm thickness were prepared using silicone molds. The size of the composite disks was prepared according to the ISO 4049:2019(E) standard. The molds were protected with a thin glass slide to avoid polymerization inhibition by oxygen. The composite disks were light‐cured at the center for 20 s and additionally at the periphery for 20 s (four overlapping zones exposed to light for 5 s each), at both sides using the LED curing light (SmartLite Pro). Therefore, the total duration of light‐curing per specimen was 80 s. Disk dimensions to the nearest 0.01 mm were measured using a digital caliper. The specimens were placed in a 37°C incubator for 22 h, then allowed to cool off to 23°C (room temperature) for 2 h and weighed to an accuracy of 0.1 mg. The specimens were maintained in a desiccator and reweighed every 24 h until a constant mass, referred to as “m1”, was reached. Specimens of each RBC disk were next immersed in 10 mL distilled water in individual glass containers for 7 days or 3 months. After that, each specimen was gently dried, waved in air for 15 s, and reweighed to an accuracy of 0.1 mg, referred to as “m2”. Then, these specimens were placed in a desiccator and dried to a stable weight, referred to as “m3”.

The water sorption (Wsp) and solubility (Wsl) in µg mm^−3^ were calculated as follows:

(1) $$\mathrm{Wsp}=\frac{m2-m3}{V}$$

(2) $$\mathrm{Wsl}=\frac{m1-m3}{V}$$

where m1 is the initial dry mass before immersion into water, m2 is the mass after immersion, and m3 is the mass that specimens have gained upon maximum water sorption, while *v* is the specimen volume.

### 4.17 Flexural Strength (FS)

The FS mechanical property was assessed by a four‐point bending test. Beam‐shaped specimens (25×2×2 mm^3^, *n* = 10 per group) of the experimental composite resins [(S_)CM‐free control RBC, 10 and 20 wt% CM‐RBC, and 10 and 20 wt% S_CM‐RBC] were produced by filling custom‐made silicone molds. For each specimen, the top and bottom surfaces were homogenously light‐cured for 60 s. Thus, per specimen underwent a total 120 s of light curing. The cured specimens were next polished with silicon carbide papers in a sequence of P1200 and P4000 (Struers; Ballerup, Denmark), and then subjected to 3, 1, and 0.5 µm diamond suspensions (Kemet International; Maidestone, UK) on cloth paper, using a polishing machine (Beta Grinder Polisher, Buehler, IL, USA). The specimens were stored for 24 h under dry conditions or for 7 days in distilled water [87]. Upon storage, the specimens were tested via an Instron universal testing machine (Instron 5848 MicroTester, Instron, Norwood, USA) in a four‐point bending test set‐up. The test was conducted at a crosshead speed of 0.5 mm/min [87]. The outer span between the supports was 20 mm, while the inner span was 10 mm (1/4 division). The FS was calculated using the following equation:

$$\Sigma =3FL/4wd^{2}$$

where *F* is the maximum load at failure, *L* is the distance between the outer supports in mm (20 mm), *w* is the width measured before testing, and *d* is the height measured before testing.

### 4.18 Degree of Conversion (DC)

FTIR spectrometer (Vertex 70v, Bruker Optik GmbH, Germany), equipped with an attenuated total reflectance crystal accessory, was used to analyze the DC of 10 and 20 wt% CM‐RBC, and 10 and 20 wt% S_CM‐RBC. The FTIR spectra for both uncured and immediately cured resin composites (*n* = 5 per group) were recorded over a wavelength range of 4000–400 cm^−1^, with a resolution of 4 cm^−1^, comprising 64 scans. Following the initial recordings, the cured specimens were stored at 37°C and retested 24 h post‐irradiation. The DC was calculated based on the ratio of peak absorption of aliphatic C═C (1638 cm^−1^) and aromatic C═C (1608 cm^−1^) before and after a 20 s irradiation period using an LED curing light (SmartLite Pro). The DC was calculated based on the following equation:

$$\mathrm{DC}\left(\%\right)=\left(1-\frac{\mathrm{Cured}\left[\frac{\mathrm{Abs}\left(1638\right)}{\mathrm{Abs}\left(1608\right)}\right]}{\mathrm{Uncured}\left[\frac{\mathrm{Abs}\left(1638\right)}{\mathrm{Abs}\left(1608\right)}\right]}\right)\times 100\%$$

### 4.19 Contact Angle and Elastic Modulus

The hydrophilic and hydrophobic properties of the resin composites (20 wt% CM‐RBC, 20 wt% S_CM‐RBC, and (S_)CM‐free control RBC formulation) were assessed through contact angle measurements, which were recorded via a Dataphysics OCA20 contact angle goniometer (DataPhysics Instruments GmbH). The elastic modulus was investigated through nanoindentation testing. Specimens measuring 7×7×2.5 mm^3^ were prepared via silicone molds. The resin composite disks (*n* = 6 per group) were light‐cured for 20 s on both sides using the LED‐curing light Bluephase N (Ivoclar Vivadent, Schaan, Liechtenstein). Finally, the disk was light‐cured for 5 s from the four sides to ensure optimal curing, resulting in a total light‐curing time of 60 s. The specimens were then stored in a dry environment for 24 h or in distilled water for 7 days. After storage, the disk was embedded in epoxy resin, sequentially wet‐ground with 1200‐ and 4000 grit SiC papers and finally polished followed using 9, 3, 1, and 0.25 µm diamond suspensions on cloth paper with a polisher (EcoMet 250, Buehler).

The nanoindentation test was performed by a quantitative nanomechanical test instrument (TI 950 TriboIndenter, Hysitron, Minneapolis, MN, USA) with a diamond indenter with an 80 nm Berkovich (three‐sided pyramidal) tip radius. A fixed force of 3000 µN and a dynamic load amplitude of 25 µN were applied [88]. The distance between adjacent points was set at 40 µm. Three specimens were analyzed for each group. The elastic modulus was derived from an average of 15 indentation points on the specimens.

### 4.20 Atomic Force Microscopy (AFM)

The surface topography of 20 wt% S_CM‐RBC and (S_)CM‐free control RBC was characterized using AFM (MultiMode 8, Bruker, Santa Barbara, CA, USA). All the mirror‐polished specimens (*n* = 3 per group) were scanned in tapping mode with a precalibrated diamond probe. The scanning area was 20 µm×20 µm and was recorded at a resolution of 256 points per line. The roughness of each specimen was analyzed with Bruker's NanoScope Analysis software.

### 4.21 Cell Culture

hDPSCs were harvested from the pulp tissues of the premolars of healthy young patients, following informed consent approved by the Institutional Review Board of Wuhan University School & Hospital of Stomatology (No. 2021A63). hDPSCs were cultured in α‐MEM added with 10% FBS and 1% penicillin/streptomycin at 37°C and 5% CO_2_.

### 4.22 Cytoskeleton Staining

Experimental resin‐composite disks (pure resin 0 wt%: (S_)CM‐free RBC, 20 wt% CM‐RBC, and 20 wt% S_CM‐RBC) with dimensions of 7×7×2.5 mm^3^ were prepared using silicone molds. Following sterilization through UV exposure, the resin‐composite disks were immersed in 5 mL of α‐MEM supplemented with 10% FBS and 1% penicillin/streptomycin for 24 h at 37°C. hDPSCs were cultured in 12‐well cell culture plates at an original density of 5×10^4^/well at 37°C. After 24 h of culturing, the culture medium was added with 500 µL fresh culture medium (Blank control) or diluted resin‐composite disk extraction. Following incubation periods of 1 day and 5 days, the cultured cells were washed with sterilized PBS and fixed with 4% paraformaldehyde. The samples were then washed with PBS, treated with a permeabilization solution for 15 min, and rinsed again with PBS. Phalloidin (Yeasen, China) and DAPI (Beyotime, China) staining were conducted to observe the morphology of hDPSCs. The samples were subsequently rinsed with PBS and sealed with an antifluorescence quenching agent (Beyotime). A fluorescence microscope (Olympus ix83, Tokyo, Japan) was used to visualize filamentous actin (stained with phalloidin, *λ*ex 488 nm) and cell nuclei (stained with 4′,6‐diamidino‐2‐phenylindole (DAPI), *λ*ex 405 nm).

### 4.23 Cytotoxicity Test by CCK‐8 Assay

hDPSCs were exposed to diluted resin‐composite disk extraction from different experimental groups. After 1 and 5‐day incubation, each well was applied with CCK‐8 (Dojindo Kagaku Co, Kumamoto, Japan) solution and cultured for 2 h at 37°C. The absorbance was determined using a microplate reader (Synergy H1, BioTek Instruments, Winooski, VT, USA) at a wavelength of 450 nm after subtracting the background. Relative cell viability (%) was set to assess the cytotoxicity of different resin‐composite disk extracts against the control group (culture medium only). The CCK‐8 assay was performed in triplicate.

### 4.24 Coomassie Blue Staining

hDPSCs were exposed to CM and S_CM powder at concentrations of 0, 5, and 10 µg mL^−1^. Following a 1‐day incubation period, the cells were stained with Coomassie blue. The cells were treated with Triton X‐100 (1%) for permeabilization, fixed with 4% paraformaldehyde, washed three times with PBS, and stained using Coomassie blue solution. Cell morphology was examined via a stereomicroscopy (Leica M125 C, Wetzlar, Germany).

### 4.25 Animals (Mice and Sprague‐Dawley Rats)

Male mice (8 weeks old with an approximate body weight of 20 g) and male Sprague‐Dawley rats (12 weeks old with an approximate body weight of 370 g) were obtained from the Center for Disease Control of Hubei Province, China. The in vivo animal experiments were conducted under the approval of the Ethics Committee of Wuhan University School & Hospital of Stomatology (No. S07924020F).

### 4.26 Toxicity Evaluation

The mice were treated with 5 or 10 µg mL^−1^ CM (within α‐MEM adding 10% FBS and 1% penicillin/streptomycin) or 5 or 10 µg mL^−1^ S_CM (within the same α‐MEM as the above) via single intraperitoneal injection in a volume of 300 µL (*n* = 5 per experimental group). After the injection, the survival rates of the mice were checked for 2 weeks. The mice injected with only α‐MEM added with 10% FBS and 1% penicillin/streptomycin were set up as the control group.

### 4.27 Blood Biochemistry Assay for Mice

After the 2‐week treatment period, the serum samples were analyzed via an automatic biochemistry analyzer equipped with specialized kits. Additionally, the concentrations of the proinflammatory cytokines TNF‐α and IL‐1β were quantitatively assessed in serum samples via kits from Elabscience Biotechnology Co., Ltd., China.

### 4.28 HE and Masson's Histological Examination

After the mice were sacrificed, the major organs, including the lung, liver, kidneys, spleen, and heart, were collected, fixed, embedded in paraffin, and sectioned. The samples were subsequently stained with hematoxylin and eosin (HE) and Masson's trichrome (Servicebio, Wuhan, Hubei, China). The slice samples were observed under the Panoramic MIDI, Panoramic250FLASH, and Panoramic DESK scanning microscope (3D HISTECH, Hungary).

### 4.29 Sprague‐Dawley Rat Tooth‐Restoration Model

Sprague‐Dawley rats were used for tooth cavity restorations (*n* = 3 per group). All the rats were maintained under specific‐pathogen‐free (SPF) conditions. SPF rats were free of *Salmonella spp*., *Corynebacterium kutscheri*, *Tyzzer's organism*, *Pasteurella pneumotropica*, *Klebsiella pneumoniae*, *Pseudomonas aeruginosa*, *Bordetella bronchiseptica*, *Mycoplasma spp*., *Hantavirus* (*HV*), *Sendai Virus* (*SV*), *Pneumonia Virus of Mice* (*PVM*), *Reovirus type III* (*Reo‐3*), *Rat Parvovirus* (*KRV*), *Rat Parvovirus* (*H‐1*), *ectoparasites*, *Toxoplasma gondii*, *all helminths*, *flagellates*, and *ciliates*. Anesthesia was induced via sodium pentobarbital (30 mg/kg) [89]. The rats were positioned on a heated surgical platform, with the maxilla and mandible secured in an open position via elastic bands to enhance access to the maxillary first molars. Initial bacteria samples were collected using oral swabs from the maxillary right and left first molars. The swabs were then placed in PBS. All teeth were then cleaned with sterilized distilled water and sodium hypochlorite (5%). Following the initial bacterial assessment, occlusal cavities were manually created in the maxillary first molars via handpieces and round diamond burs, with dimensions of 1 mm in depth and 1 mm in width. Afterward, the cavity was applied with Single Bond Universal (3M Oral Health, USA) in self‐etch mode. The adhesive was light‐cured for 10 s using the LED‐curing light (Bluephase N). Finally, the experimental resin composites (10 and 20 wt% S_CM‐RBC) and the (S_)CM‐free control RBC were applied separately into the cavities.

After the restorations, the rats were orally infected with 200 µL of *S*. *mutans ATCC 25175* (10^9^ CFU mL^−1^) twice daily and were provided with a cariogenic diet (Keyes 2000, comprising 56% powdered sucrose to facilitate the implantation of *S*. *mutans*) along with cariogenic water. To prevent the adverse effects associated with continuous anesthesia, oral infections were administered on the first, fourth, seventh, and 14th days (twice daily) postcomposite restorations. On day 14, just prior to the fourth infection, viable *S*. *mutans* bacteria were collected from the maxillary right and left first molars using oral swabs, and the bacterial cell numbers were determined. On day 21, before the rats were euthanized, *S*. *mutans* was collected from the first molars using the previously described method.

### 4.30 DNA Extraction and Sequencing Analysis

The initial bacteria, as well as the bacteria after 14 and 21 days of treatment on the maxillary first molars were suspended in 500 µL PBS. DNA extraction was performed with a bacterial DNA kit according to the manufacturer's instructions. The qPCR was performed with ChamQ Universal SYBR qPCR Master Mix (Nanjing Vazyme Biotech Co.). Relative quantification and fold changes were evaluated by the 2^−ΔΔCt^ method. Universal primers specific for bacterial 16S rRNA genes [81] and *S*. *mutans* primers (targeting the *gtfB* gene) were used.

### 4.31 Rat Blood Biochemistry Assay

Prior to euthanizing the rats, blood samples were collected via cardiac puncture. The serum samples were then analyzed by an automatic biochemistry analyzer equipped with kits, and a routine blood examination was conducted by an automatic blood cell analyzer.

### 4.32 Histological Analyses

After the rats were sacrificed, the maxillae were harvested and fixed in a 4% paraformaldehyde phosphate buffer solution before being decalcified in a 10% ethylenediamine tetraacetic acid solution prepared in PBS. After decalcification, the samples were dehydrated in ethanol solutions, infiltrated with paraffin, and sectioned at a thickness of 5 µm. Furthermore, the sections were stained with HE and Masson's trichrome staining (Servicebio, Wuhan, Hubei, China). The gingival tissues were stained with HE. The prepared slices were observed under Panoramic MIDI, Panoramic250FLASH, and Panoramic DESK scanning microscopes (3D HISTECH, Hungary).

### 4.33 Statistical Analysis

The data are presented as mean ± SD, with the number of samples (*n*) indicated in the text or figure legends. One‐way ANOVA with Tukey's post hoc pairwise comparisons or Dunnett's multiple comparisons was used to check for statistical significance. When the data exhibited unequal variance, Welch's ANOVA with Games‐Howell contrast was used to perform the statistical analysis using SPSS software (version 26.0; SPSS, Chicago, IL, USA). Regarding FS, DC, nanoelastic modulus, and roughness data, statistical analysis was conducted using two‐way ANOVA with interaction. When the *p*‐value is less than 0.05, it is considered statistical different, with *p < 0.05, **p < 0.01, ***p < 0.001, and ****p < 0.0001, indicating varying significance levels.

## Conflicts of Interest

The authors declare no conflicts of interest.

## Supporting information




**Supporting File 1**: advs75146‐sup‐0001‐SuppMat.docx.

**Supplemental Movie 1**: advs75146‐sup‐0002‐Movie S1.mov.

**Supplemental Movie 2**: advs75146‐sup‐0003‐Movie S2.mov.

## Data Availability

The data that support the findings of this study are available from the corresponding author upon reasonable request.
