# Supplementary material for: Advanced Dental Composite Technology via Bisilanized Dual‐Action Nanofillers for Biofilm Control
Source: Adv Sci (Weinh). 2026 Apr 7;13(36):e75146. doi: 10.1002/advs.75146 (PMC13317598; doi:10.1002/advs.75146)
Supplement: Supplementary file 1 — Supporting File 1: advs75146‐sup‐0001‐SuppMat.docx. [file ADVS-13-e75146-s002.docx]

**Supporting Information**

**Advanced Dental Composite Technology via Bi-silanized Dual-action Nanofillers for Biofilm Control**

*Chenmin Yao, Line Etiennot, Naiera Zayed, Shengjie Liang, Mehraveh Saghi, Jelle Verdonck, Lingyue Liu,* *Fei Zhang, Wim Teughels, Cui Huang, Kirsten Van Landuyt, Bart Van Meerbeek**

C. Yao

KU Leuven, Department of Oral Health Sciences, BIOMAT & UZ Leuven, Dentistry, Leuven 3000, Belgium

State Key Laboratory of Oral & Maxillofacial Reconstruction and Regeneration, Key Laboratory of Oral Biomedicine Ministry of Education, Hubei Key Laboratory of Stomatology, School & Hospital of Stomatology, Wuhan University, Wuhan 430079, China

L. Etiennot, K. Van Landuyt, B. Van Meerbeek

KU Leuven, Department of Oral Health Sciences, BIOMAT & UZ Leuven, Dentistry, Leuven 3000, Belgium

Email: bart.vanmeerbeek@kuleuven.be

N. Zayed, M. Saghi, W. Teughels

KU Leuven, Department of Oral Health Sciences, Periodontology & Oral Microbiology, Leuven 3000, Belgium

S. Liang, C. Huang

State Key Laboratory of Oral & Maxillofacial Reconstruction and Regeneration, Key Laboratory of Oral Biomedicine Ministry of Education, Hubei Key Laboratory of Stomatology, School & Hospital of Stomatology, Wuhan University, Wuhan 430079, China

J. Verdonck

KU Leuven, Department of Public Health and Primary Care, Environment and Health, Leuven 3000, Belgium

L. Liu

KU Leuven, Department of Chemical Engineering, Soft Matter, Rheology and Technology (SMaRT), Leuven 3001, Belgium

F. Zhang

KU Leuven, Department of Oral Health Sciences, BIOMAT & UZ Leuven, Dentistry, Leuven 3000, Belgium

KU Leuven, Department of Materials Engineering, Surface and Interface Engineered Materials (SIEM), Leuven 3001, Belgium

The Supporting Information contains

- Figures S1-S14,
- Tables S1-S5,
- Movies S1-S2.

**
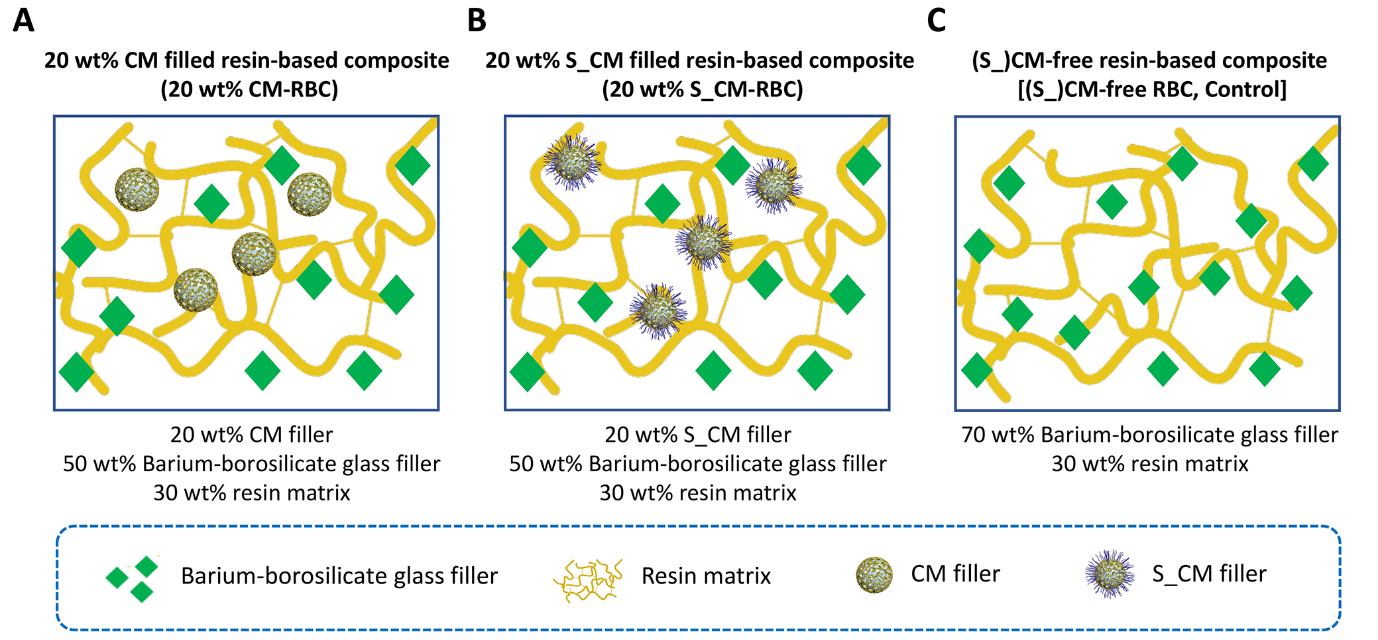
**

**Figure S1.** Component proportions within the experimental resin-based composite (RBC) formulations. (A) 20 wt% CM-RBC, (B) 20 wt% S_CM-RBC and (C) (S_)CM-free control RBC.

**
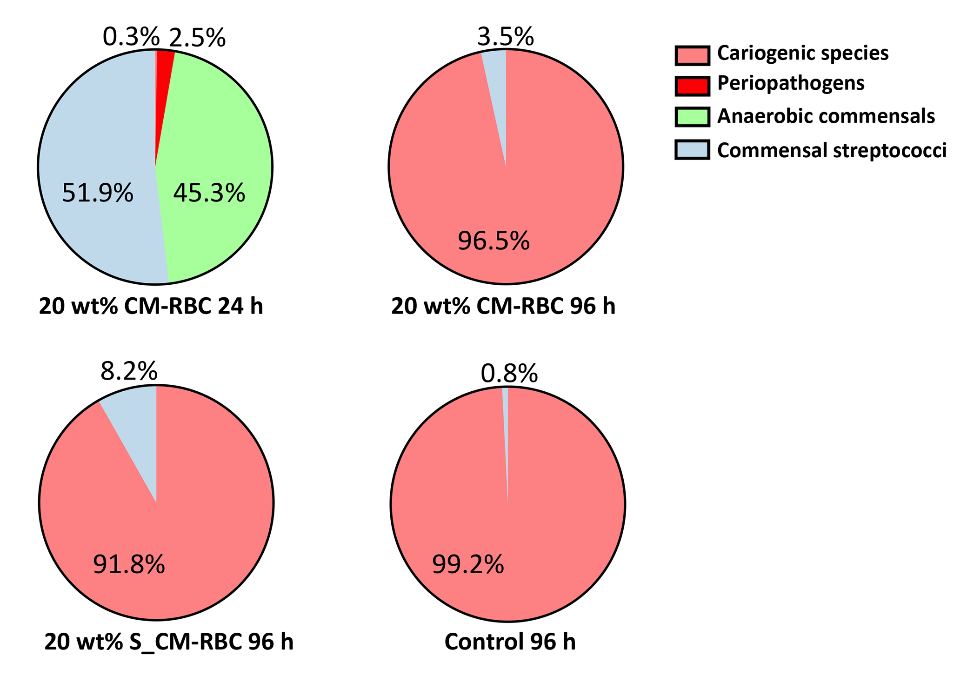
**

**Figure S2.** Proportional composition of the 24 h biofilm of 20 wt% CM-RBC and the 96 h biofilm of 20 wt% CM-RBC, 20 wt% S_CM-RBC and (S_)CM-free control RBC. Biofilms are divided into cariogenic species, periopathogens, anaerobic commensals and commensal streptococci.

**
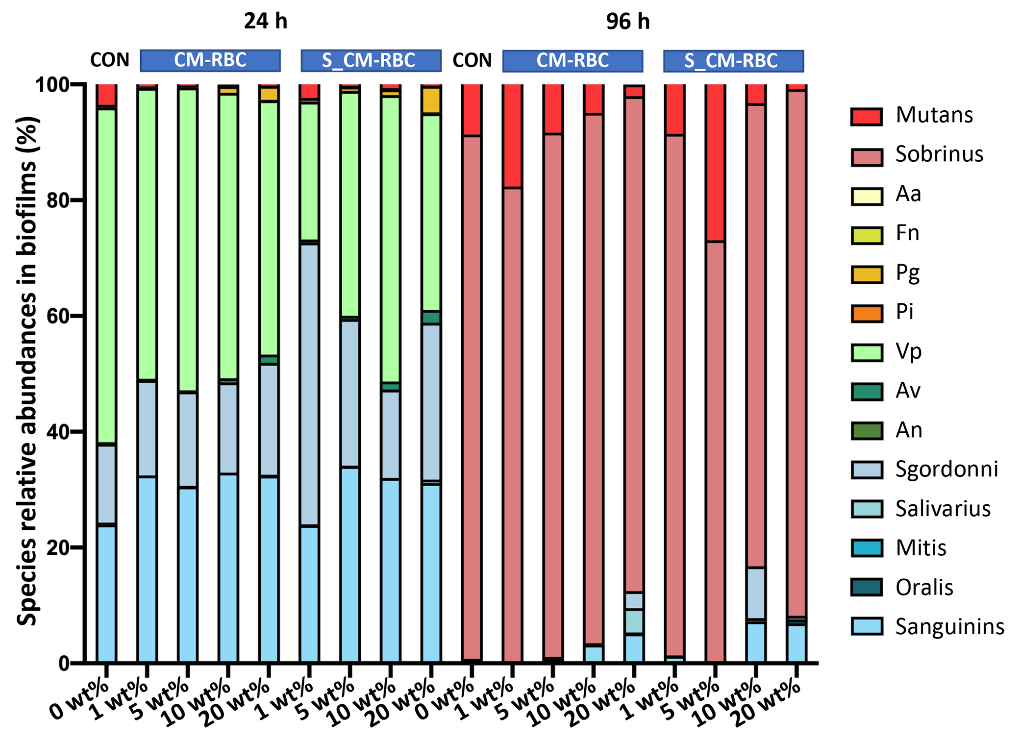
**

**Figure S3.** Composition of the biofilms (species relative abundances) following different RBC treatments, as determined using species-specific primers. Mutans: *Streptococcus mutans*, Sobrinus: *Streptococcus sobrinus*, Aa: *Aggregatibacter actinomycetemcomitans*, Fn: *Fusobacterium nucleatum*, Pg: *Porphyromonas gingivalis*, Pi: *Prevotella intermedia*, Vp: *Veillonella parvula,* Av: *Actinomyces viscosus,* An: *Actinomyces naeslundii*, Sgordonni: *Streptococcus gordonii*, Salivarius: *Streptococcus salivarius*, Mitis: *Streptococcus mitis*, Oralis: *Streptococcus oralis,* Sangguinins: *Streptococcus sanguinis*.

**
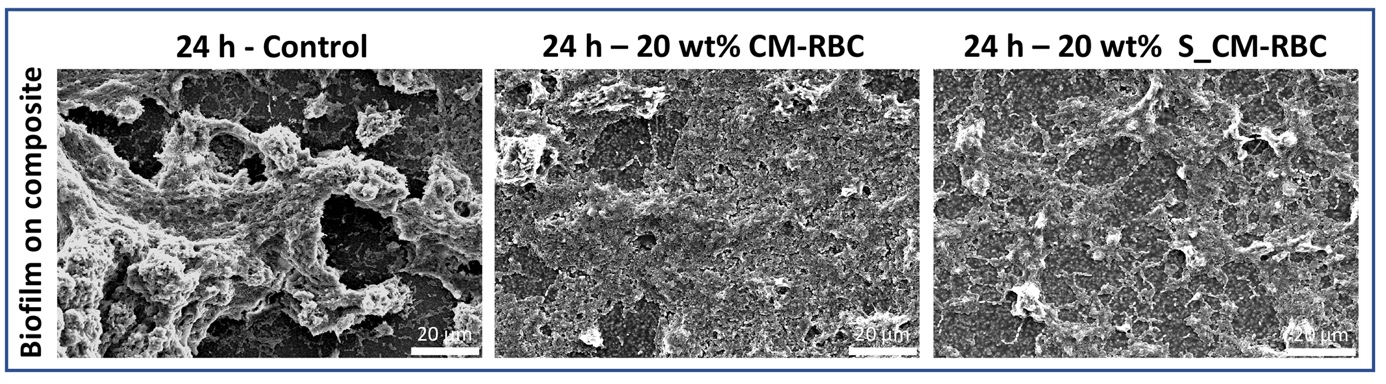
**

**Figure S4.** Low-magnification SEM photomicrographs of bacterial adhesion and 24 h biofilm deposition on a composite disk for the (S_)CM-free control and on composite disks filled with 20 wt% CM and 20 wt% S_CM filler, referred to as 20 wt% CM-RBC and 20 wt% S_CM-RBC.

**
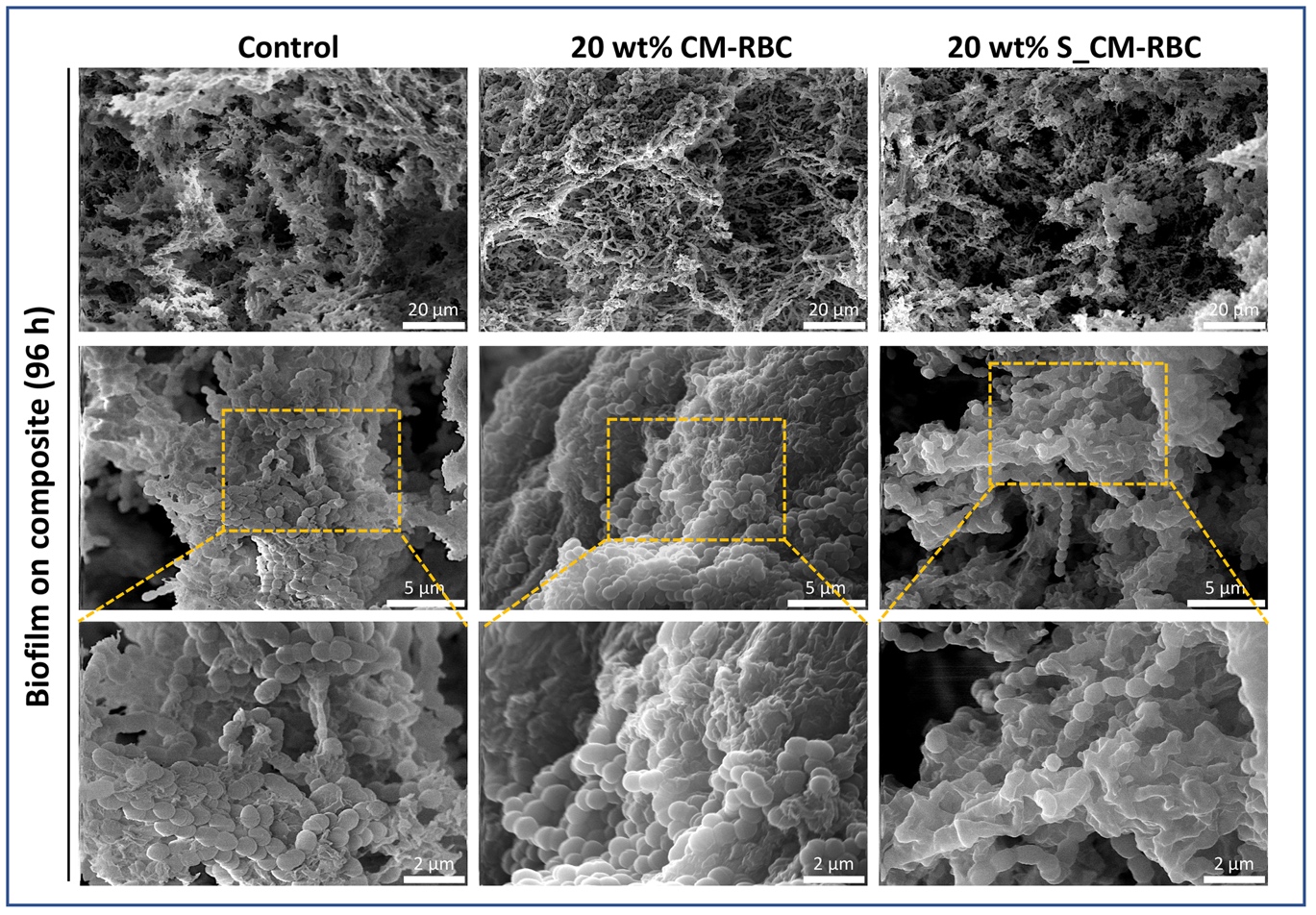
**

**Figure S5.** SEM photomicrographs of bacterial adhesion and 96 h biofilm deposition on a composite disk for the (S_)CM-free control and on composite disks filled with 20 wt% CM and 20 wt% S_CM filler, referred to as 20 wt% CM-RBC and 20 wt% S_CM-RBC.

**
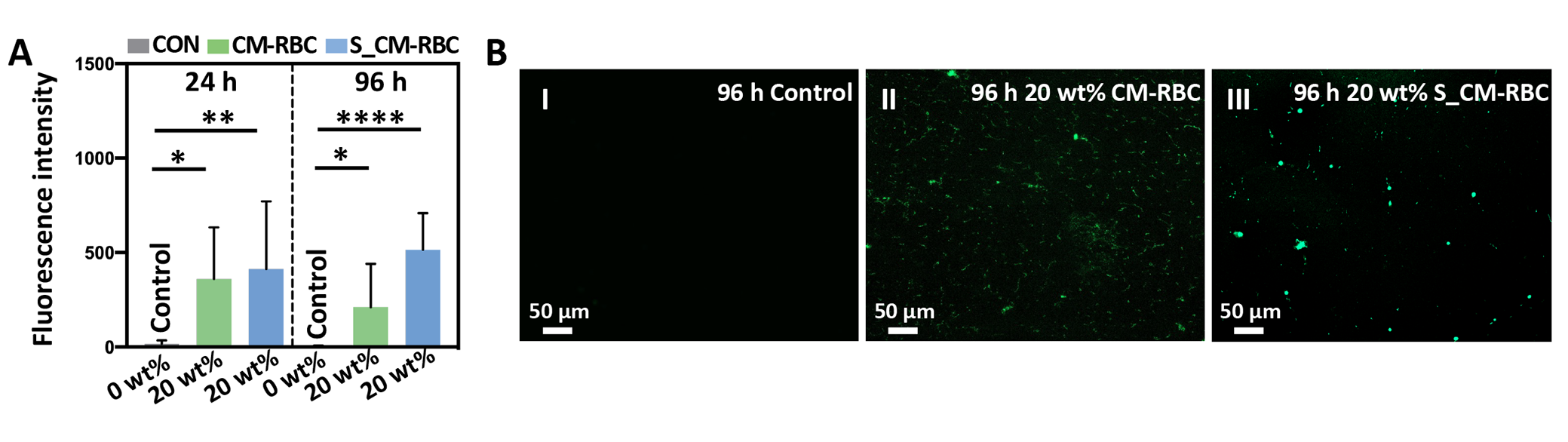
**

**Figure S6.** Intracellular ROS generation of S. mutans treated with S_CM-RBC. (A) Intracellular ROS expression of bacterial communities (24 or 96 h) after CM-RBC and S_CM-RBC treatment based on green fluorescence intensity. Two biological replicates with five fields of view each were analyzed. One-way ANOVA with Dunnett's multiple comparisons test: **p <* 0.05, ***p <* 0.01, and *****p <* 0.0001. (B) The green fluorescence of DCF indicates that 20 wt% S_CM-RBC treatment results in higher ROS production within the bacteria after 96 h of treatment.

**
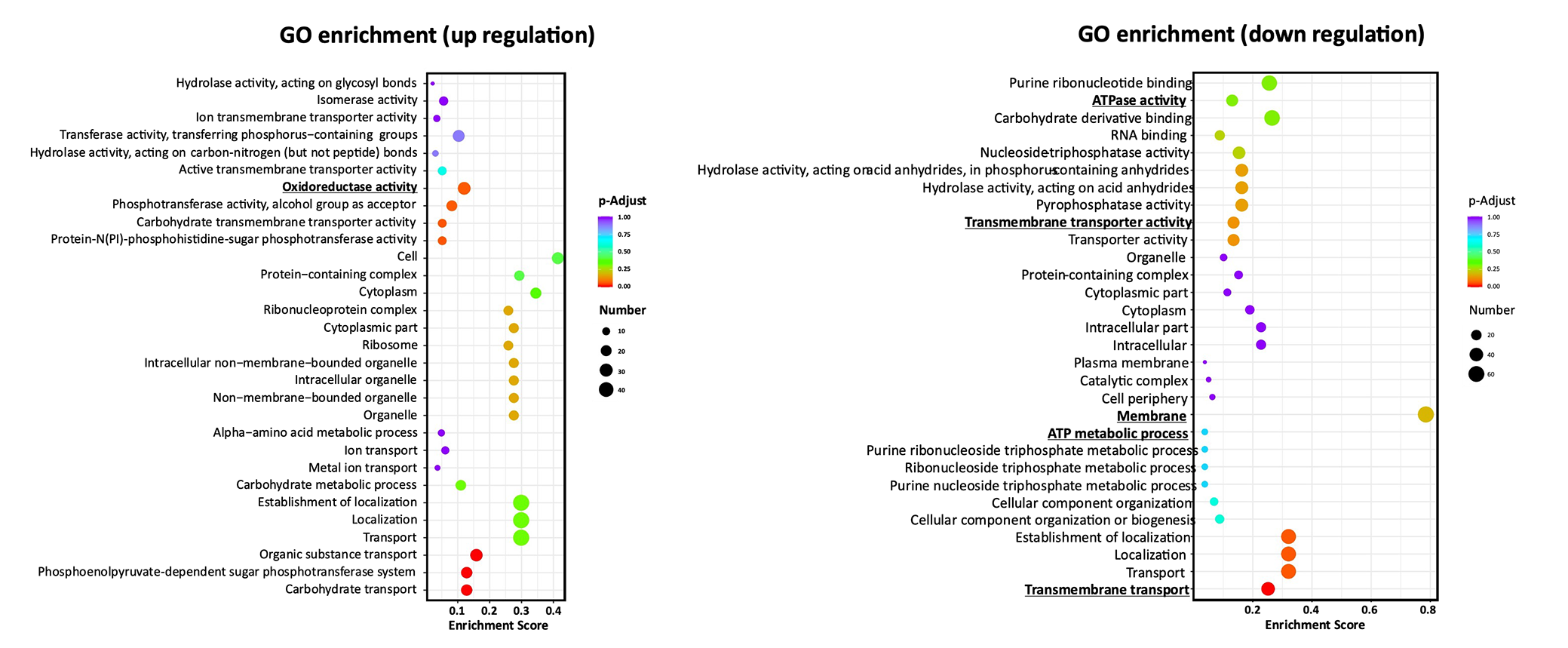
**

**Figure S7.** GO enrichment analysis of upregulated and downregulated DEGs in the S_CM treated group compared to the control group.

**
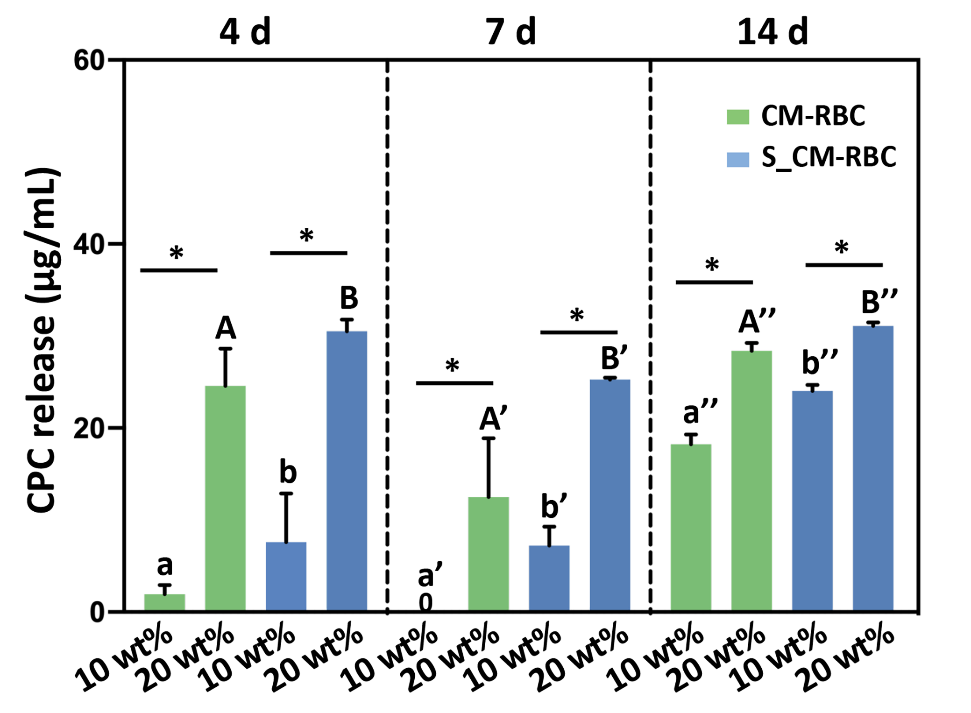
**

**Figure S8.** CPC release from 10 and 20 wt% CM-RBC (green bars), and from 10 and 20 wt% S_CM-RBC (blue bars) after 4, 7 and 14 days. Data are expressed as means ± SD, with the same lower or upper letters indicating absence of statistical difference in pairwise comparisons within the category of CM and S_CM (*p* > 0.05, n = 5), and with columns marked with an asterisk being significantly different in pairwise comparisons within concentration (**p <* 0.05, Two-way ANOVA).

**
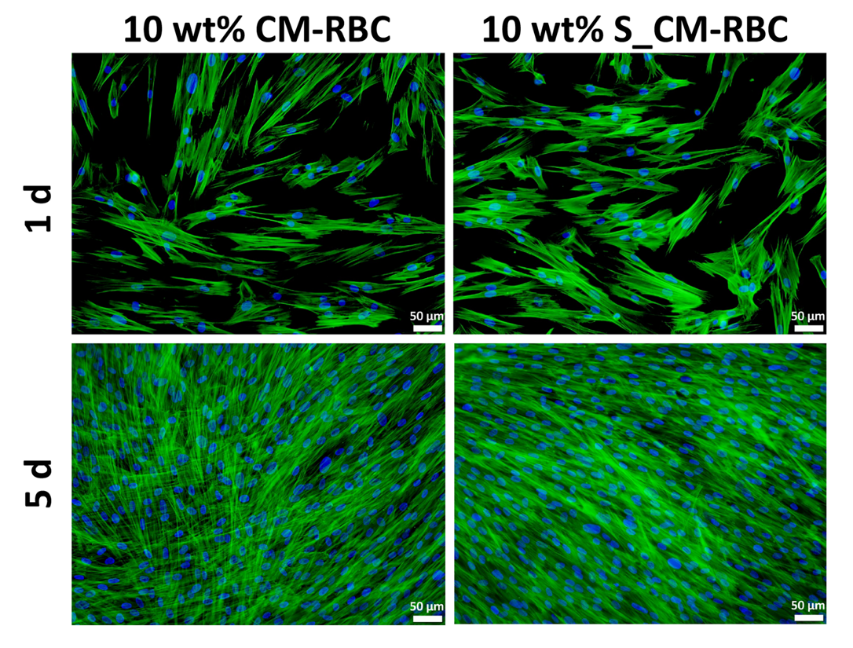
**

**Figure S9.** Fluorescent labeling of the nucleus (blue) and actin (green) in hDSPCs at 1 and 5 days after incubation with diluted resin-composite disk extraction (10 wt% CM-RBC and 10 wt% S_CM-RBC).

**
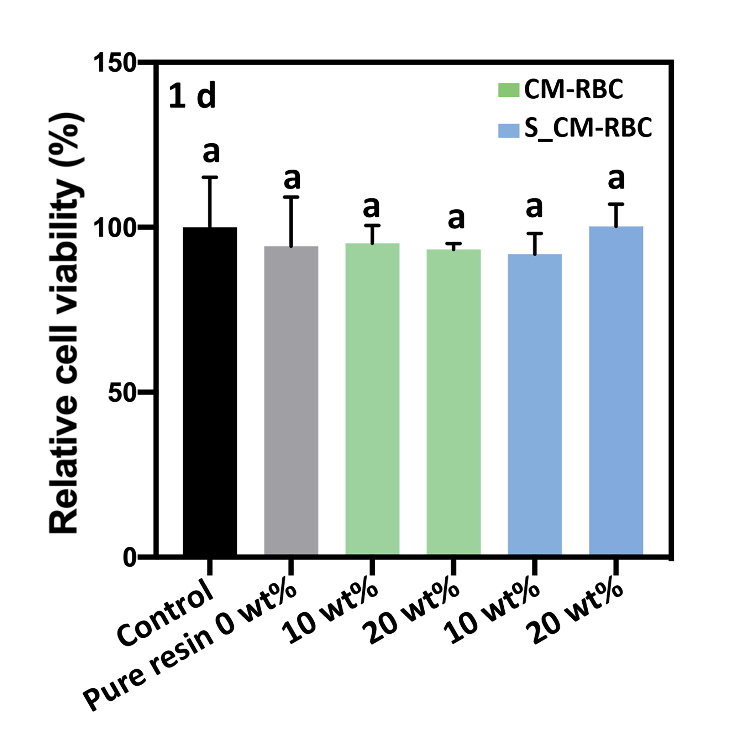
**

**Figure S10.** Cell viability of hDSPCs cultured with resin-composite disk extraction for 1 day and analyzed by a CCK-8 assay (Control: α-MEM with 10% FBS and 1% penicillin/streptomycin added; Pure resin 0%: (S_)CM-free RBC). Data are expressed as means ± SD. One-way ANOVA with Tukey’s test. The same small letters indicate the absence of statistical difference (*p* > 0.05, n = 3).

**
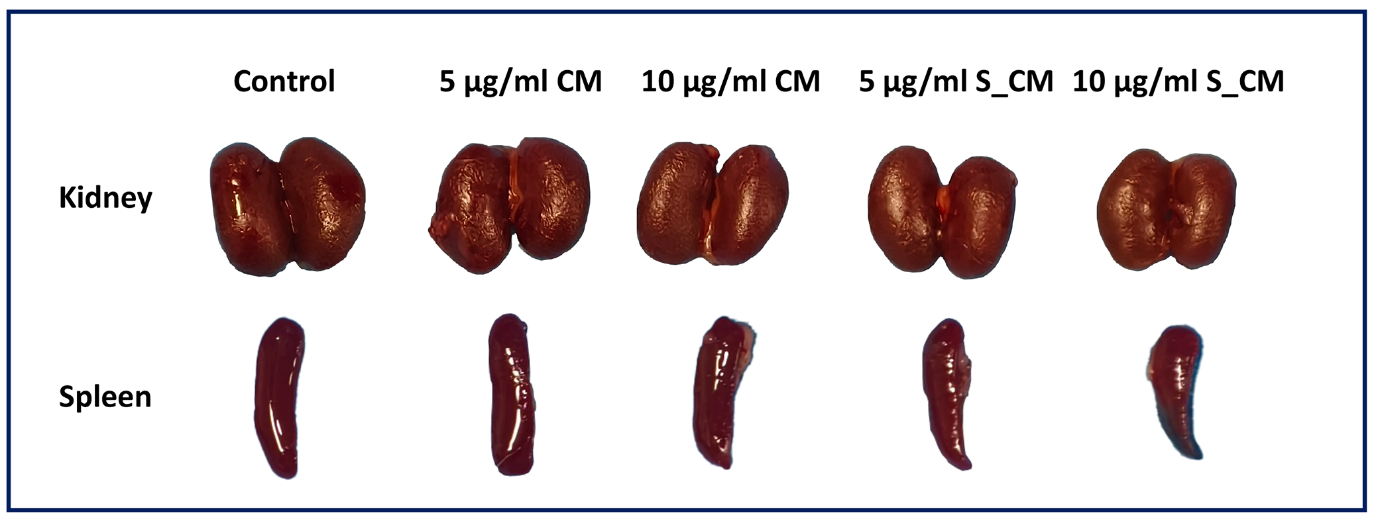
**

**Figure S11.** Representative images of the kidneys and spleens of mice subjected to different treatments (Control: following injection of α-MEM with 10% FBS and 1% penicillin/streptomycin added via a single intraperitoneal injection).

**
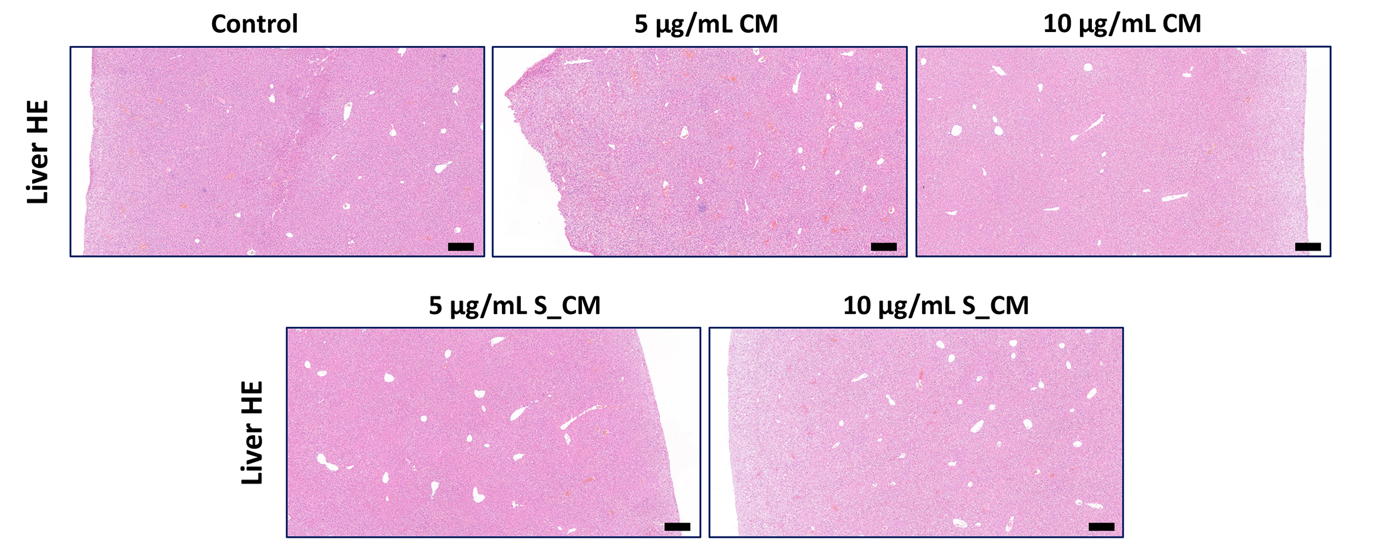
**

**Figure S12.** Histological examination (HE staining) of the liver from mice upon single intraperitoneal administration of 5 and 10 µg/mL CM and S_CM. Scale bars: 200 µm (low magnification).

**
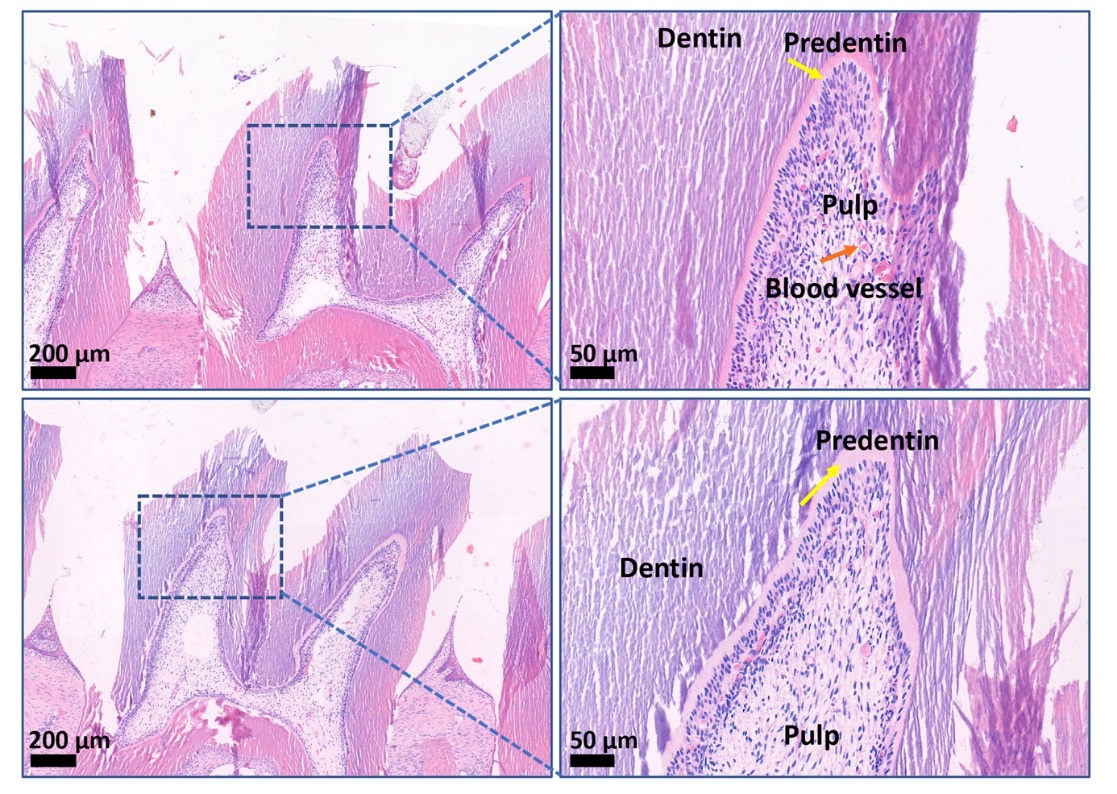
**

**Figure S13.** HE staining of non-restored second molars (control) from rats revealed healthy pulp-tissue conditions. Yellow arrows indicate predentin. The orange arrow refers to blood vessels.

**
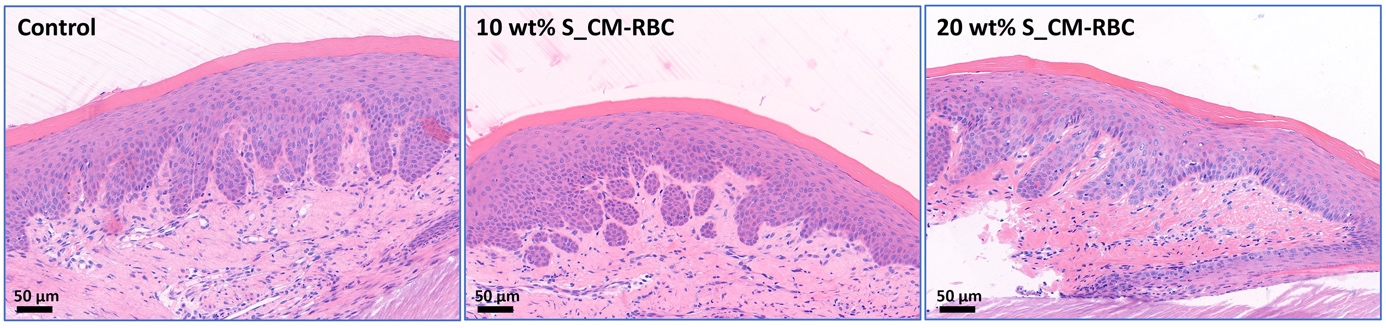
**

**Figure S14.** Histopathology of rat gingival tissues on day 21 following treatment with the (S_)CM-free control RBC, and the experimental 10 and 20 wt% S_CM-RBC.

**Supplementary Tables**

**Table S1**. Filler-particle properties of MSN, CPC@MSN (CM), and S_CPC@MSN (S_CM).

| **Materials** | **S_BET_**  **(m^2^/g)** | **D_DFT_**  **(nm)** | **Vp-_DFT_**  **(cc/g)** | **Vt-pore volume (cc/g)** |
| --- | --- | --- | --- | --- |
| MSN | 675.6 | 3.2 | 0.415 | 0.421 |
| CM | 72.0 | 6.0 | 0.088 | 0.079 |
| S_CM | 50.5 | 4.9 | 0.056 | 0.026 |

S_BET_: specific surface area; D_DFT_: pore diameter using Density Functional Theory (DFT) calculation; Vp-_DFT_: total pore volume using DFT calculation; Vt-pore volume: single point pore volume calculated from the adsorption isotherm.

**Table S2**. Component proportions (in wt%) within the experimental resin-composite formulations.

| **Resin-composite formulations** | **S_CM** | **Silanized barium-borosilicate glass filler** | **Resin matrix** |
| --- | --- | --- | --- |
| Control [(S_)CM-free RBC] | 0 | 70 | 30 |
| 1 wt% S_CM-RBC | 1 | 69 | 30 |
| 5 wt% S_CM-RBC | 5 | 65 | 30 |
| 10 wt% S_CM-RBC | 10 | 60 | 30 |
| 20 wt% S_CM-RBC | 20 | 50 | 30 |

**Table S3**. Bacterial strains used in this study.

| **PATHOBIONTS** | | **COMMENSALS** | |
| --- | --- | --- | --- |
| **Cariogenic pathogens** | **Periodontal**  **pathogens** | **Anaerobic**  **commensals** | **Streptococci commensals** |
| *Streptococcus mutans*  ATCC 20523  *(S. mutans)* | *Aggregatibacter actinomycetemcomitans*  ATCC 43718 (*Aa*) | *Actinomyces naeslundii*  ATCC 51655 *(An)* | *Streptococcus gordonii* ATCC 49818 |
| *Streptococcus sobrinus*  ATCC 20742  *(S. sobrinus)* | *Fusobacterium nucleatum* DSM 20482 (*Fn*) | *Actinomyces viscosus* DSM 43327 *(Av)* | *Streptococcus mitis* DSM 12643 |
|  | *Porphyromonas gingivalis* ATCC 33277 (*Pg*) | *Veillonella parvula*  DSM 2008 *(Vp)* | *Streptococcus oralis* DSM 20627 |
|  | *Prevotella intermedia*  ATCC 25611 (*Pi*) |  | *Streptococcus salivarius* TOVE-R |
|  |  |  | *Streptococcus sanguinis* LM 14657 |

**Table S4**. Bacterial strains, primers and probes used.

| **Species** | **Primer/probe 5’→3’** | **Product size (bp)** | **Target** |
| --- | --- | --- | --- |
| *Streptococcus*  *mutans* | F: GCC TAC AGC TCA GAG ATG CTA TTC T  R: GCC ATA CAC CAC TCA TGA ATT GA  P: TGG AAA TGA CGG TCG CCG TTA TGA A | 114 | *gtfB* gene |
| *Streptococcus*  *sobrinus* | F: AAA TAC GGC CAG TGC CAA AG  R: CCA GCC TGA GAT TCA GCT TGT  P: CCT GCT CCA GCG ACA AAG GCA GC | 165 | *gtfT* gene |
| *Aggregatibacter actinomycetemcomitans* | F: CGG TGT CGA TTT GGG GAT TGG  R: TGC AGC ACC TGT CTC AAA GC  P: AGA ACT CAG AGA TGG GTT TGT GCC TTA GGG | 237 | 16S rRNA gene |
| *Fusobacterium nucleatum* | F: GGA TTT ATT GGG CGT AAA GC  R: ATC TGT CCA GTA AGC TGG CTT CC  P: CTC TAC ACT TGT AGT TCC G | 191 | 16S rRNA gene |
| *Porphyromonas gingivalis* | F: CCG TAA GAA TAA GCA TCG GCT AAC TC  R: CAC GAA TTC CGC CTG C  P: CAC TGA ACT CAA GCC CGG CAG TTT CAA | 195 | 16S rRNA gene |
| *Prevotella*  *intermedia* | F: TGT GCC CYT TTG CAT TTA CCC TTC  R: CAC CAT GAA TTC CGC ATA CG  P:TGG CGG ACT TGA GTG CAC GC | 216 | 16S rRNA gene |
| *Actinomyces*  *naeslundii* | F: TCG AAA CTC AGC AAG TAG CCG  R: AGA GGA GGG CCA CAA AAG AAA  P: GGG TAC TCT AGT CCA AAC TGG CGG ATA GCG | 96 | gene encoding unknown protein |
| *Actinomyces*  *viscosus* | F: GTG AAG GAG CCA GCT TGC TGG TTC TG  R: CGG AAC AAA CCT TTC CCA GGC  P: ATG AGT GGC GAA CGG GTG AGT AAC | 155 | 16S rRNA gene |
| *Streptococcus*  *gordonii* | F: GAA GAA CTG GGT AGC GAT TGC T  R: GTT AGC TGT TGG ATT GGT TGC C  P: AGA ACA GTC CGC TGT TCA GAG CAA | 262 | *gtfG* gene |
| *Streptococcus*  *mitis* DSM 12643 | F: GGC TCG TAG TCT GGA GAT GG  R: TAG GTC GTC GTC CCA AGG AA  P: CGA AGA GCA CCA ATA GCA CCT CCC | 133 | 16S rRNA gene |
| *Streptococcus*  *oralis* DSM 20627 | F:ACC AGC AGA TAC GAA AGA AGC AT  R: AGG TTC GGG CAA GCG ATC TTT CT  P: AAG GCT GCT GTT GCT GAA GAA GT | 229 | *gtfR* gene |
| *Streptococcus*  *salivarius* | F: GAC GAT GAC TGT CAA CTT GAC AC  R: ACC GTA ACG TGG GAA AAC TG  P: GTA GCG TCA GAG TGG TTG AC | 247 | Dextranase gene |
| *Streptococcus*  *sanguinis* LMG 14657 | F: CAA AAT TGT TGC AAA TCC AAA GG  R: GCT ATC GCT CCC TGT CTT TGA  P: AAA GAA AGA TCG CTT GCC AGA ACC GG | 75 | *gtfP* gene |
| *Veillonella parvula*  DSM 2008 | F: GAC GAA AGT CTG ACG GAG CA  R: TGC CAC CTA CGT ATT ACC GC  P: AGC TCT GTT AAT CGG GAC GAA AGG C | 171 | 16S rRNA gene |

F: forward primer; R: reverse primer; P: probe; bp: base pair.

**Table S5**. Ingredients of the qPCR mixture (25 μL).

| **Ingredient** | **Quantity** |
| --- | --- |
| Master Mix  Sterile Milli-Q water  Forward primer  Reverse primer  Probe  DNA template | 12.5 μl  4.5 μl  1 μl  1 μl  1 μl  5 μl |
